# Learning-dependent 4 Hz synchronization in the posterior striatum, lateral geniculate nucleus, and visual cortex

**DOI:** 10.1016/j.isci.2025.113958

**Published:** 2025-11-06

**Authors:** Sai Tanimoto, Shigeyoshi Fujisawa

**Affiliations:** 1 Laboratory for Systems Neurophysiology, RIKEN Center for Brain Science, Wako, Saitama 351-0198, Japan; 2 Department of Complexity Science and Engineering, Graduate School of Frontier Sciences, the University of Tokyo, Kashiwa, Chiba 277-0882, Japan

**Keywords:** Natural sciences, Biological sciences, Neuroscience, Behavioral neuroscience, Sensory neuroscience

## Abstract

The posterior striatum (pStr) is thought to play a crucial role in perceptual learning by maintaining stable sensory representations based on past experience and associating them with appropriate motor responses. However, how the pStr neuronal activity acquires these representations, in particular the functional significance of the interactions between the pStr and its sensory input regions, remains largely unexplored. Here, we conducted simultaneous and chronic electrophysiological recordings from the pStr, dorsolateral geniculate nucleus, and visual cortex in rats performing a visual discrimination task. We found that learning enhanced 4 Hz synchronization between the pStr and visual areas, especially during decision-making periods. Moreover, following learning, regular-spiking neurons in the pStr exhibited increased firing rates and stronger phase-locking to these oscillations. These results highlight the involvement of the pStr in visual perceptual learning and indicate the role of 4 Hz oscillations within sensory-striatal circuits in facilitating sensorimotor integration required for efficient task performance.

## Highlights

• The posterior striatum (pStr) changes activity patterns during visual learning
• Learning enhances 4 Hz synchronization between the pStr and visual areas
• The enhanced 4 Hz synchronization is specific to the task phase
• Learning increases the proportion of the pStr neurons phase-locked to 4 Hz

## Graphical abstract

**Figure:**
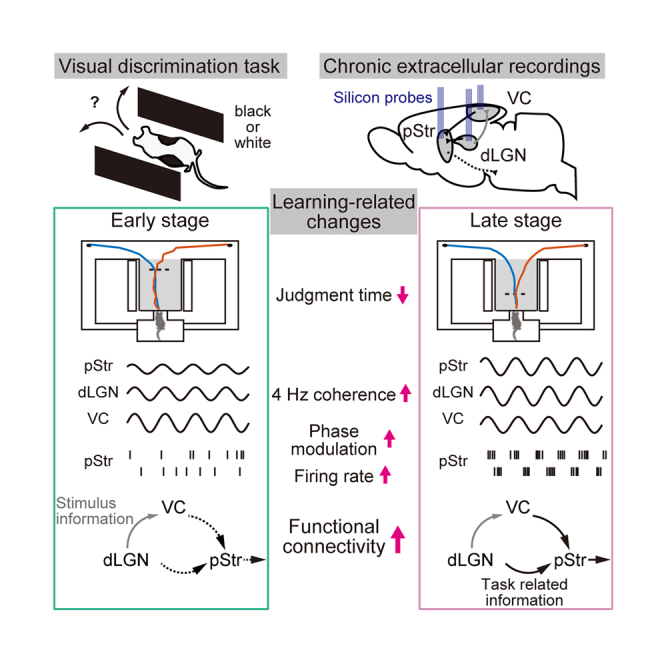

## Introduction

Perceptual decision-making requires the integration of sensory information into motor planning and execution. While sensory discrimination learning has traditionally been associated with cortical and thalamic structures, recent evidence suggests that the posterior striatum (pStr) plays a key role in linking sensory inputs with appropriate motor responses.^1^^,^^2^^,^^3^^,^^4^^,^^5^ In contrast to the anterior striatum, which has been extensively studied in action selection and reinforcement learning, the pStr is uniquely positioned to process sensory-driven behaviors via its dense innervation from sensory cortices and thalamic nuclei.^6^^,^^7^^,^^8^^,^^9^^,^^10^^,^^11^ Studies in primates, including humans, indicate that the caudate tail, a region homologous to the rodent pStr, contains stable sensory representations linked to past experience.^12^^,^^13^^,^^14^^,^^15^^,^^16^^,^^17^^,^^18^^,^^19^^,^^20^^,^^21^^,^^22^^,^^23^^,^^24^^,^^25^ However, the functional role of the pStr in sensory learning, particularly its interactions with sensory cortices and thalamic nuclei, remains unclear.

To date, most studies demonstrating learning-dependent changes in activity in the pStr have employed auditory paradigms, despite the existence of innervations to the pStr from visual areas.^3^^,^^5^^,^^26^^,^^27^ By contrast, research on visual discrimination has primarily focused on the canonical visual pathway, where retinal inputs are relayed from the dorsolateral geniculate nucleus (dLGN) to the visual cortex (VC).^28^^,^^29^^,^^30^^,^^31^ This leaves open the question of how the pStr contributes to visual discrimination learning and interfaces with established visual circuits. In particular, the development and evolution of neuronal synchronization between these areas over the course of learning remains poorly understood, primarily due to the technical challenges associated with achieving stable and long-term extracellular recordings from the same regions.

Neuronal oscillations provide an essential mechanism for coordinating distributed activity across brain regions. Low-frequency rhythms, in particular, can temporally organize neuronal spiking and facilitate long-range communication.^32^ Among them, 4 Hz oscillations have been implicated in synchronizing striatum and other structures, such as prefrontal cortex, to support task-specific information processing.^33^^,^^34^^,^^35^^,^^36^^,^^37^ Disruptions of these rhythms have been linked to executive dysfunction in disorders such as Parkinson’s disease.^36^^,^^38^^,^^39^^,^^40^ Conversely, abnormally persistent or enhanced 4 Hz oscillations have been implicated in the reactivation and recurrence of maladaptive memories in psychiatric conditions such as addiction, obsessive-compulsive disorder, and post-traumatic stress disorder.^37^ Despite these insights, most studies have focused on the ventral or anterior striatum and their interactions with frontal cortices. In contrast, whether such oscillations are also involved in communication between the pStr and sensory areas remains unknown.

To investigate the role of the pStr and its interaction with visual areas in visual discrimination learning, we trained rats on a T-maze in which visual cues instructed left or right choices while simultaneously and chronically recording neuronal activity from the pStr, dLGN, and VC. By applying hierarchical clustering to the behavioral metrics, we defined learning stages and examined learning-dependent changes in the pStr neuronal firing patterns. We then analyzed local field potentials (LFPs) to quantify inter-regional coherence and phase-locking, with a focus on 4 Hz oscillations. Our results shed light on the role of the pStr in perceptual decision-making and its functional connectivity with visual processing regions.

## Results

### Progressive learning process of the visual discrimination task

**Figure 1:**
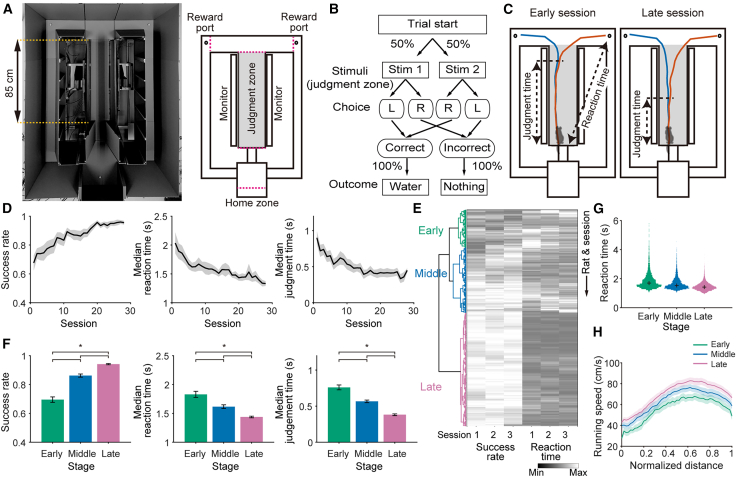
Learning process in a visual discrimination task (A and B) Schematic of a visual discrimination task. The T-shaped maze apparatus (A) and the task procedure (B) are shown. Visual stimuli (white or black; denoted as Stim 1 and 2, respectively) were presented on the monitors placed on both sides of the central arm (judgment zone; shaded in the right panel (A)). Five infrared sensors were installed along the maze to detect the time of rat passage (indicated by dotted magenta lines; right panel (A)). Rats discriminated the color of the monitors and chose either the left or the right arm. If the rats chose the correct arm, they received a water reward on 100% of trials. Otherwise, no reward was delivered. (C) Example trajectories of a rat from an early session and a late session. The bifurcation point at which trajectories differed significantly between left- and right-choice trials was defined as the judgment point (*p* < 0.05, permutation test). Judgment time was the time from entry into the judgment zone (A) to the judgment point. Reaction time was the time from entry into the judgment zone to arrival at each reward port. (D) Success rate, reaction time, and judgment time in the learning process across sessions (*N* = 13 rats). Mean (black line) ± SEM (shaded). (E) Dendrogram based on success rate and reaction time computed over consecutive sets of three sessions (*N* = 349 session sets). Sessions were partitioned into three stages: early, middle, and late. (F) Averages of the success rate, reaction time, and judgment time for each learning stage (*N* = 13 rats). Vertical bars represent mean ± SEM. ∗: *p* < 0.05 (one-way ANOVA followed by Wilcoxon rank-sum test). (G) Distributions of reaction times across learning stages. Black crosses denote the median of the distributions. (H) Averaged running-speed profiles aligned to normalized distance within the judgment zone at the early (green), middle (blue), and late (purple) learning stages. Shaded areas represent ±SEM. See also Figure S1.

Rats were trained to perform a visual discrimination task in a T-shaped maze, where they were required to choose the left or right arm based on visual cues presented on monitors in the central arm (“judgment zone”) (Figures 1A and 1B). The judgment zone was designed to be wide so that we could detect the bifurcation point of the rats’ trajectory toward the left or right target (Figure 1A). We trained the rats for approximately one month to track the behavioral changes (28.9 sessions on average). The rats’ head positions were monitored by a camera set on the ceiling and analyzed using DeepLabCut.^41^ We found that the rats’ trajectories initially bifurcated near the end of the central arm, but as learning progressed, the bifurcation points occurred at earlier positions (Figure 1C). Behavioral analyses showed a gradual improvement in task performance over training sessions, as indicated by an increase in the success rate. In addition, reaction time and judgment time decreased as training progressed, suggesting improved task efficiency (Figure 1D).

To categorize learning stages in an unsupervised manner, we applied hierarchical clustering to sequences of the success rate and reaction time separated into three consecutive sessions. This analysis identified three distinct learning stages: (1) an early stage characterized by a low success rate and long reaction time, (2) a middle stage with intermediate performance, and (3) a late stage where the success rate was high and reaction time was short (Figure 1E). Alongside the increase in session indexes from early to late learning stages (Figure S1), the temporal evolution of the behavioral parameters across these stages tracked the learning curve (Figures 1D and 1F). In parallel, the distribution of reaction time gradually changed, as reflected by the decrease in skewness (Figure 1G; skewness values of 2.42, 1.94, and 1.43, in the early, middle, and late learning stages, respectively). Together, these observations support the validity of the learning phase classification. Consistent with the shortening of the reaction time, running speed increased as learning progressed, and this increase was uniform across all positions within the judgment zone (Figure 1H).

### Increased posterior striatum regular-spiking neuronal activity in the judgment zone after learning

We then investigated the neuronal networks underlying this improvement in visual discrimination learning and task efficiency. We hypothesized that the pStr would be involved, given its innervation from multiple sensory regions, including the visual thalamus (dLGN) and visual cortex (VC),^8^^,^^10^ and its role in maintaining stable representations of past experiences.^3^^,^^5^^,^^13^^,^^18^ Since previous studies relied on anterograde axonal labeling or retrograde tracing, leaving open the possibility that they labeled the passing fibers, we first asked whether the pStr neurons directly receive axonal inputs from the dLGN and VC. Using AAV1, which enables the *trans*-synaptic expression of Cre or FlpO, we found evidence consistent with projections from both the dLGN and VC onto the pStr neurons (Figure S2). These findings support the view that the pStr is a pivotal node for improving task efficiency during learning.

**Figure 2:**
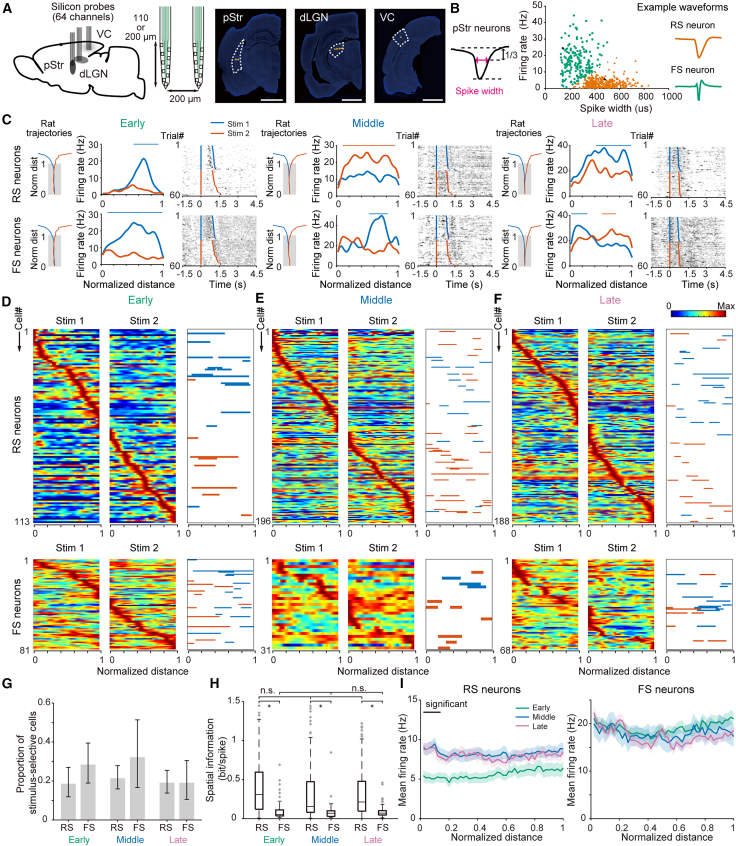
Neuronal activity of the pStr during the visual discrimination task and its evolution across learning (A) Schematic of the silicon probe implantation. Silicon probes (inset) were implanted in the pStr, dLGN, and VC (left). Sections with the electrode tracks were stained with DAPI (right). Arrowheads represent the electrode tips. Scale bars: 2.5 mm. Summary of electrode locations is shown in Figure S3. Neuronal activities of the dLGN and VC are shown in Figures S6 and S7, respectively. (B) Classification of regular-spiking (RS; orange triangles) and fast-spiking (FS; green circles) neurons in the pStr by spike width and firing rate. Spike width was defined as the duration for which waveform amplitude remained below one-third of the trough-to-peak amplitude (left). Example waveforms for each cluster are shown on the right (the corresponding units are highlighted in black in the middle panel). (C) Firing patterns of representative RS (top) and FS (bottom) neurons at the early, middle, and late learning stages. Left, trajectories of the rats for the sessions in which the representative neurons were recorded. Blue and red lines indicate trajectories when the rats chose the left or right arm, respectively. Shaded areas represent the judgment zone. “Norm dist” indicates normalized distance. Middle, average firing rate as a function of position in the judgment zone in Stim 1 (blue) and 2 (red) trials, respectively. Bars above the plots represent the locations where the unit activities were significantly different between Stim 1 and 2 (*p* < 0.05, permutation test). Right, spike raster plots as a function of time for each trial, sorted by stimulus type. The first blue or red lines represent the time the rats entered the judgment zone, and the second lines represent the time the rats exited the judgment zone, followed by the monitors turning gray. (D–F) Firing patterns of pStr RS (top) and FS (bottom) neurons in Stim 1 (first column) and 2 (second column) trials during the early (D), middle (E), and late (F) learning stages. The color scale represents the firing rate of each neuron (the first and second columns). The third column shows locations with a significantly higher discharge rates in Stim 1 (blue) or 2 (red) trials (*p* < 0.05; permutation test). (G) Proportion of stimulus-selective cells in the pStr RS and FS populations at each learning stage. Vertical bars represent the 95% confidence intervals (Clopper-Pearson method). (H) Spatial information in pStr RS and FS populations at each learning stage (∗: *p* < 0.05, two-way ANOVA). (I) Averaged firing rate of pStr RS (left) and FS (right) neurons at the early (green), middle (blue), and late (purple) learning stages. Shaded areas represent ±SEM. Horizontal bars above the plots represent the location with a significantly higher firing rate between early and late sessions (*p* < 0.05; permutation test). See also Figures S4 and S5.

Next, we recorded extracellular single unit activity from the pStr, dLGN, and VC using high-density silicon probes during the early, middle, and late learning stages. In total, we recorded 689 well-isolated units from the pStr, 995 units from the dLGN, and 526 units from the VC in 13 rats (Figures 2A and S3; Table S1). We first focused on the neuronal activity in the pStr. Striatal neurons are typically classified into two major subtypes: medium spiny neurons (MSNs), which are projection neurons of this region, and interneurons, which form local inhibitory connections.^33^^,^^42^^,^^43^^,^^44^ To distinguish between neuronal subtypes, we classified the pStr neurons into two subtypes based on their spike width and firing rate: regular-spiking (RS) neurons, corresponding to putative MSNs, and fast-spiking (FS) neurons, corresponding to putative interneurons^43^^,^^44^^,^^45^^,^^46^^,^^47^^,^^48^ (Figure 2B). Since the number of tonically active neurons, corresponding to cholinergic interneurons, was small (only 10 neurons across all recordings; Figure S4A),^49^ we grouped them with putative PV interneurons and treated them as FS neurons for subsequent analyses. FS neurons constituted 26.6% of our sample, which is considerably higher than the reported anatomical proportion (∼5%).^50^ Previous studies identifying FS neurons in the striatum have reported sampling biases,^45^^,^^46^^,^^51^^,^^52^ suggesting that this disparity arises from the fact that FS neurons, due to their high firing rates, are more easily detected and stably clustered during spike sorting.^46^ Furthermore, autocorrelograms for RS and FS neurons in our dataset were consistent with those reported in previous studies^46^^,^^48^ (Figure S4B). We therefore consider our classification to be valid.

To assess stimulus- or position-selective firing activities of the pStr neurons in the task, we analyzed the discharge activities of individual neurons across different conditions during single sessions in the early, middle, and late learning stages (Figures 2C–2F). Trials were labeled by the presented stimulus identity: Stim 1 (white) and 2 (black). A subset of neurons exhibited different firing patterns between the stimulus types when the rats ran in the judgment zone. To investigate trial type-specific firing in single neurons, we applied a permutation-based statistical test to compare the position-dependent firing rates in the different trial types^53^ (see STAR Methods). This approach allowed us to identify neurons that discharged differentially between Stim 1 and 2 trials (Figures 2D–2F, the rightmost column). Approximately 20% of the pStr neurons (23.7%, 23.0%, and 18.8% in the early, middle, and late stages, respectively) exhibited significantly different firing rates between Stim 1 and 2 trials (*p* < 0.05, permutation test), indicating their involvement in visual discrimination. The proportion of stimulus-selective cells did not differ significantly between RS and FS neurons or across the learning stages (Figure 2G).

Next, we calculated mutual information based on the position occupancy^54^ to evaluate the spatial information carried by these neurons. Overall, RS neurons exhibited significantly greater spatial information than FS neurons (median 0.21 vs. 0.06, *p* < 0.01, Wilcoxon rank-sum test), with 71.8% of RS neurons and 28.3% of FS neurons having spatial information greater than 0.1 bit/spike. This trend was consistent across learning stages. Additionally, within each neuronal subtype, spatial information did not vary significantly across learning stages or between correct movement directions (Figures 2H and S5A, two-way ANOVA). Regardless of the position preference in individual neurons, the population of neurons fired relatively uniformly across the entire judgment zone, independent of subtypes (Figures 2D–2F). Furthermore, when comparing trials in which the correct choice was ipsilateral versus contralateral to the recording hemisphere, the distribution of the average firing rates during traversal of the judgment zone showed no significant bias toward either side (Figure S5B, D’Agostino’s skewness test, skewness values of 0.016, 0.951, and 0.641 for RS neurons, and 0.001, 0.007, and 0.032 for FS neurons in the early, middle, and late learning stages, respectively).

Finally, to examine the population-level activity changes over learning, we averaged RS and FS activities. RS neuron activity significantly increased in the late learning stage, particularly at the entry to the judgment zone, where the rats engaged in decision-making (Figure 2I). In contrast, FS neuron activity remained stable throughout the learning stages. Thus, two major differences between the neuronal subtypes emerged: RS neurons exhibited stronger position selectivity than FS neurons, which is stable across learning stages, and only RS neurons showed position-specific increased activity in the late learning stage.

We also examined the neuronal activity in the dLGN and VC during the task across learning stages to determine whether the pStr features reflected their inputs. In the VC, units were classified into putative excitatory neurons (RS neurons) and inhibitory interneurons (FS neurons) based on their spike width and firing rate^55^^,^^56^^,^^57^^,^^58^ (Figures S7A and S7B). The proportion of putative inhibitory neurons was 19.4%, which was consistent with previous studies.^57^^,^^58^ By contrast, we did not subdivide dLGN units into RS and FS. Anatomically, interneurons comprise only ∼5% of dLGN neurons, and in our recordings, no units satisfied the prior spike-width criteria for FS neurons (Figures S6A and S6B).^57^^,^^58^^,^^59^ A sizable number of neurons in the dLGN and VC showed trial-type-dependent firing during the task (Figures S6C–S6E and S7C–S7E), as observed in the pStr. Notably, the proportion of stimulus-selective neurons was much higher in the dLGN and VC than that in the pStr (Figures 2G, S6F, and S7F). Where subtype classification was feasible, FS neurons were more stimulus-selective than RS neurons (pStr and VC; Figures 2G and S7F). Regarding spatial selectivity, RS neurons carried significantly more spatial information than FS neurons in the VC, with no stage-dependent change within subtype, similar to the pStr neurons (Figures 2H and S7G; two-way ANOVA). By contrast, the dLGN spatial information (pooled) increased with learning (Figure S6G; one-way ANOVA). Overall, the fraction of neurons with spatial information greater than 0.1 bit/spike was much higher in the pStr compared to the dLGN and VC (57.9%, 16.1%, and 35.9%, respectively). At the population level, dLGN activity decreased significantly across learning, whereas VC population activity remained unchanged (Figures S6H and S7H; permutation test).

### Enhanced 4 Hz coherence between regions in the late learning stage

**Figure 3:**
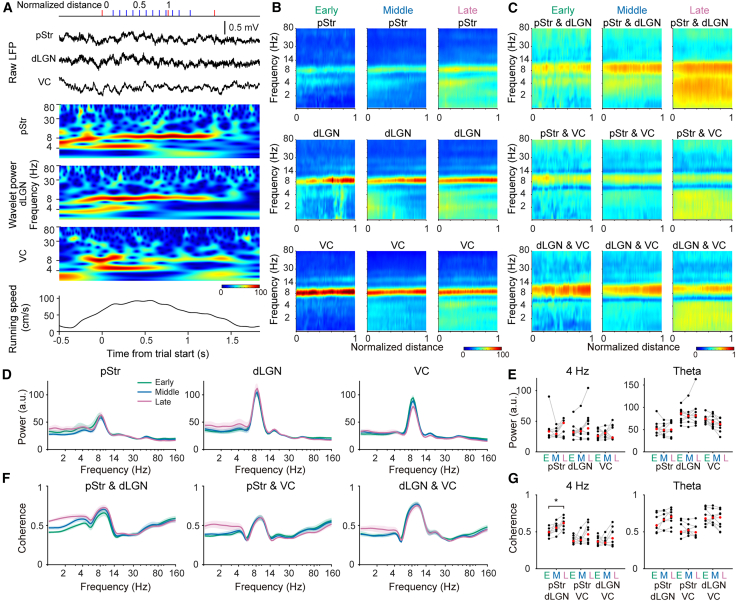
Enhanced 4 Hz coherence between the pStr and dLGN after learning (A) Representative LFP traces and corresponding wavelet power spectra in the pStr, dLGN, and VC. Running speed is also shown (bottom). Red ticks at the top mark the trial onset, stimulus offset, and choice times. Blue lines represent the normalized positions in the maze. (B) Average wavelet power as a function of position for example rat pStr (top), dLGN (middle), and VC (bottom) in the early (left), middle (middle), and late (right) sessions (*N* = 2, 6, and 8 sessions, respectively). The color scale represents the wavelet power. (C) Average wavelet coherence for the same rat as in (B) in the early (left), middle (middle), and late (right) sessions. pStr vs. dLGN (top), pStr vs. VC (middle), and dLGN vs. VC (bottom) are shown. The color scale represents the wavelet coherence. (D) Average wavelet power in the judgment zone (*N* = 9 rats for pStr and *N* = 10 rats for dLGN and VC). Green, blue, and purple lines represent the early, middle, and late stages, respectively. Shaded areas represent ±SEM. (E) Mean 4 Hz (left, 2–5 Hz) and theta (right, 6–10 Hz) wavelet power for each region and learning stage. Black dots represent individual rats, and red dots represent the group mean. (F) Average wavelet coherence within the judgment zone (*N* = 8 rats for pStr vs. dLGN and pStr vs. VC pairs and *N* = 9 rats for dLGN vs. VC). Green, blue, and purple lines represent the early, middle, and late stages, respectively. Shaded areas represent ±SEM. (G) Mean 4 Hz (left, 2–5 Hz) and theta (right, 6–10 Hz) wavelet coherence for each region pair and learning stage. Black dots represent individual rats, and red dots represent the group means. ∗: *p* < 0.05 (one-way ANOVA followed by Wilcoxon signed rank test). See also Figures S8–S11.

Having characterized the local response properties, we next assessed how these dynamics are coordinated at the network level. To this end, we analyzed simultaneously recorded local field potentials (LFPs) from the pStr, dLGN, and VC in 11 rats to examine inter-regional interactions across learning. During the judgment-zone period, two prominent oscillatory activities were observed in all three regions: theta (6–10 Hz) and 4 Hz (2–5 Hz) oscillations (Figures 3A–3C). To identify which frequency band was associated with learning stages, we compared the wavelet power in the judgment zone across learning stages (Figures 3B, 3D, and 3E) and found that both 4 Hz and theta band power remained relatively stable (Figure 3E).

Next, we examined inter-regional coherence in the judgment zone across learning stages (Figures 3C, 3F, and 3G). This analysis revealed a significant increase in 4 Hz coherence between the pStr and dLGN in the late learning stage compared to the early stage (Figures 3G and S8, *p* < 0.05, Wilcoxon signed rank test). In contrast, neither the coherence in the 4 Hz band between other region pairs nor in the theta band between any region pairs changed significantly across learning stages (Figure 3G). To determine whether the observed increase in 4 Hz coherence between the pStr and dLGN was specific to the judgment zone, we also analyzed coherence during other task phases when the rats occupied different maze locations. In these control phases, neither the 4 Hz nor theta power or coherence showed significant stage-dependent changes for any pair of regions (Figure S9). When aligned to normalized distance, 4 Hz power was consistently high at the judgment-zone entry and declined as the rats moved forward, irrespective of the learning stage. In contrast, theta power exhibited region-specific patterns. In the pStr, it peaked at a normalized distance of 0.2–0.4, whereas in the dLGN and VC it increased toward the end of the judgment zone (Figure S10A). 4 Hz coherence remained relatively stable along the spatial axis of the judgment zone but increased overall across learning stages, while theta coherence changed little with learning (Figure S10B). Additionally, 4 Hz coherence did not differ significantly between success and failure trials (Figure S10C, two-way ANOVA).

To address whether these oscillations were associated with behavioral parameters such as speed, we calculated Kendall’s tau coefficient between the amplitude of these oscillations and the running speed of the rats (Figure S11). Theta oscillations in all regions showed strong positive correlations with running speed (median coefficient: 0.20, 0.40, and 0.35 in the late stage for the pStr, dLGN, and VC oscillations, respectively), whereas 4 Hz oscillations exhibited only weak correlations (median coefficient: −0.07, −0.10, and 0.02 in the late stage for the pStr, dLGN, and VC oscillations, respectively). The coefficients did not change significantly across the learning stages (Figure S11D; one-way ANOVA). These results indicate that theta oscillations in these regions are closely linked to behavioral parameters such as running speed, whereas 4 Hz oscillations may reflect internal processes such as decision preparation and learning-dependent network reorganization.

### Phase-locking of posterior striatum neurons to 4 Hz oscillations in the late learning stage

**Figure 4:**
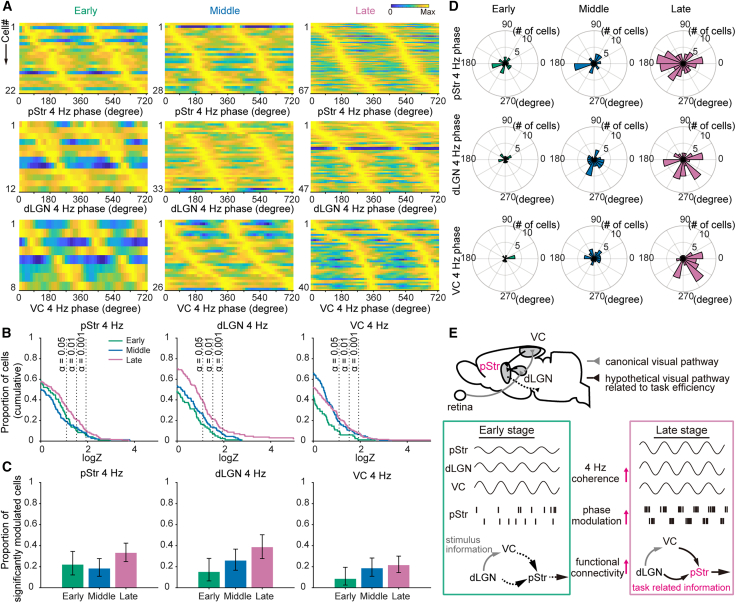
Entrainment of the pStr neuronal firing by 4 Hz oscillations (A) Phase modulation of the pStr neuron firing with respect to 4 Hz oscillations referenced to the pStr (top), dLGN (middle), and VC (bottom) during the early (left), middle (middle), and late (right) learning stages. Only significantly phase-modulated neurons are shown (*p* < 0.05, Rayleigh test). The color scale represents the normalized firing rate of each neuron. The proportions of significantly phase-modulated neurons (early, middle, and late) were as follows: pStr-referenced, 22.9%, 18.9%, and 33.8%; dLGN-referenced, 22.9%, 22.3%, and 23.7%; and VC-referenced, 8.3%, 17.6%, and 20.2%. (B) Cumulative density function of phase modulation strength statistics log Z (Rayleigh Z statistic) for the pStr neurons. Green, blue, and purple lines represent the early, middle, and late stages, respectively. (C) Proportion of the pStr neurons phase-locked to 4 Hz oscillations referenced to the pStr (left), dLGN (middle), and VC (right) at each learning stage. Vertical bars represent the 95% confidence intervals (Clopper-Pearson method). (D) Histograms of preferred phase for the pStr neurons with respect to pStr (top), dLGN (middle), and VC (bottom) 4 Hz oscillations at the early (left), middle (middle), and late (right) learning stages. (E) Schematic of the hypothetical visual pathways conveying task-relevant information. After learning, the 4 Hz coherence between the pStr and visual areas increases, and a larger fraction of the pStr neurons is phase-modulated by visual-area 4 Hz oscillations. This strengthens functional connectivity between the pStr and visual areas, enabling rapid information transfer to downstream targets and improving task efficiency.

Finally, we investigated how the increase in 4 Hz coherence in the late learning stage influenced firing activity in these regions. We found that a substantial proportion of neurons in the pStr were significantly modulated by the 4 Hz oscillation (Figure 4A; Rayleigh test), and the proportion increased significantly at the late stage (Figures 4B and 4C; Kolmogorov-Smirnov test). To assess the phase preferences of these neurons, we analyzed the firing phases relative to each region’s 4 Hz oscillation. At the early learning stage, preferred phases were broadly distributed. However, at the late learning stage, neurons phase-locked to the dLGN or VC 4 Hz oscillations converged on similar preferred firing phases around 300°, corresponding to the peak of the 4 Hz cycle. In contrast, neurons phase-locked to the pStr 4 Hz oscillation preferred phases are around 180°, corresponding to the trough (Figures 4A and 4D). These results indicate that learning may enhance the 4 Hz synchronization between the visual areas and the pStr, promoting strengthened functional coupling across the network (Figure 4E).

## Discussion

In this study, we investigated the neuronal mechanisms underlying visual discrimination learning in rats and found that 4 Hz synchronization between the pStr and visual areas may enhance their functional connectivity and support high task efficiency. We first identified three distinct learning stages based on behavioral performance. As learning progressed, rats adjusted their trajectories earlier and showed improved task efficiency, as reflected by higher success rates and shorter reaction times. Neuronal recordings revealed a significant increase in the activity of pStr RS neurons during the late learning stage, suggesting a role in task execution. In addition, we observed enhanced 4 Hz coherence between the pStr and dLGN, accompanied by the increased phase-locking of pStr neurons to 4 Hz oscillations. Collectively, these findings suggest that the pStr contributes to perceptual decision-making during visual discrimination, with 4 Hz synchronization facilitating efficient communication between visual processing areas and the pStr after learning.

### Task-phase specific increase in firing rates of posterior striatum regular-spiking neurons after learning

Accumulating evidence suggests that the pStr plays a specialized role in perceptual decision-making by integrating sensory inputs with contextual expectations and modulating response thresholds.^1^^,^^2^^,^^3^^,^^4^^,^^5^ Anatomical studies have revealed that the pStr receives abundant inputs from sensory related areas, including both the thalamus and neocortex, suggesting functional segregation from other subregions of the striatum.^6^^,^^7^^,^^8^^,^^9^^,^^10^ In contrast to the anterior dorsal striatum, the optogenetic activation of direct-pathway neurons in the pStr does not elicit movement outside task contexts. Instead, it biases decisions toward the contralateral side and specifically influences choices during the sensory evidence accumulation phase. These findings support the view that the pStr is selectively engaged in task contexts and is crucial for linking sensory information to specific reward-driven actions.^1^^,^^2^^,^^3^^,^^4^^,^^5^^,^^60^^,^^61^^,^^62^^,^^63^

Electrophysiological recordings in rodents have revealed that the pStr neurons reliably represent sensory stimuli even after learning, independent of subsequent decisions,^3^ with similar findings in non-human primates^13^^,^^14^^,^^21^ and humans.^23^^,^^24^ Cross-species studies further indicate that the pStr, or the caudate tail in primates, is a conserved hub for sensory-value integration. In primates, the caudate tail receives strong inputs from higher-order visual cortical areas and primarily processes visual information, whereas in rodents it serves as a multimodal hub integrating visual, auditory, and somatosensory inputs.^11^^,^^17^ In both cases, the pStr links sensory representations with stable value signals to bias behavior, though the dominant sensory domain differs across species. Within this broader framework, our current results add a new dimension by demonstrating that RS neurons in the rodent pStr exhibit a learning-dependent enhancement of activity at the onset of the judgment zone, coinciding with improved behavioral performance. This task-phase-specific modulation suggests that learning dynamically strengthens the sensory-action linkage in the pStr, allowing stable sensory representations to be selectively amplified when they are most relevant to decision formation. In other words, the pStr does not merely store value information but can adaptively increase its influence on downstream circuits as the behaviors become more proficient, supporting the emergence of robust, habit-like decision strategies.

The striatum contains two major interneuron subtypes: parvalbumin-positive (PV) and cholinergic interneurons.^49^^,^^64^^,^^65^^,^^66^ PV interneurons in the pStr provide feedforward inhibition to both direct- and indirect-pathway MSNs, regulating the responsiveness of these neurons to incoming inputs and maintaining a balance between the two competing pathways.^5^^,^^27^^,^^42^^,^^67^^,^^68^ Although the role of cholinergic interneurons in the pStr is poorly understood, in general, they are thought to encode stimulus salience and modulate the speed of learning.^61^ In our study, FS neurons carried less spatial information than RS neurons, and their activities remained stable across learning stages. Because of limitations inherent to extracellular recording techniques, cholinergic interneurons were only rarely isolated in our recordings, and we were unable to analyze them separately. Nevertheless, our results are consistent with prior work indicating that inhibitory interneurons primarily serve to maintain network stability in the perceptual decision-making process.

### Task-phase specific increase in 4 Hz coherence between the posterior striatum and dorsolateral geniculate nucleus after learning

Recent studies have highlighted the importance of the pStr in perceptual decision-making, yet the source of its incoming information remains unclear. In our study, anatomical tracing confirmed that the pStr receives direct inputs from both the dLGN and VC, consistent with prior reports identifying the pStr as a convergence hub for sensory information.^7^^,^^8^^,^^9^^,^^10^ Based on these anatomical connections, we simultaneously recorded LFPs from the pStr, dLGN, and VC and found pronounced 4 Hz coherence between the pStr and dLGN when the rats traversed the judgment zone during the late learning stage.

Previous work suggests that 4 Hz oscillations synchronize activities between the medial prefrontal cortex and ventral tegmental area during working memory and interval timing tasks, linking cortical inputs with striatal outputs to support task-specific information processing.^33^^,^^34^^,^^35^^,^^36^ Impairments in these 4 Hz oscillations have been implicated in neurological disorders such as Parkinson’s disease, where patients often exhibit impairments in executive control. In such cases, the attenuation of medial frontal 4 Hz oscillations correlates with cognitive dysfunction.^40^ Furthermore, depleting cortical dopamine neurons or overexpressing D2 receptors in the striatum reduces 4 Hz oscillations, suggesting that these oscillations rely on intact dopaminergic systems, including the frontal cortex and the mesolimbic system.^36^^,^^38^^,^^39^^,^^40^ Conversely, stimulating frontal dopamine neurons at this frequency can compensate for impaired action timing control or cognitive processing in both dopamine-depleted rodents and patients with Parkinson’s disease.^69^^,^^70^^,^^71^

Despite these findings, most studies on 4 Hz oscillations have focused on the ventral or anterior striatum, and their functions outside of fronto-striatal circuits remain largely unexplored. In general, low frequency oscillations such as 4 Hz are thought to connect distributed neuronal circuits by synchronizing convergent inputs.^32^ Indeed, recent work has shown that VTA-derived 4 Hz rhythms can organize beta-band synchrony across distributed brain regions, including the prefrontal cortex, nucleus accumbens, amygdala, and hippocampus, supporting a broader role of 4 Hz oscillations in coordinating large-scale memory- and cognition-related networks.^37^ In line with this mechanism, we observed that both 4 Hz synchronization between the pStr and visual areas and the proportion of the pStr neurons phase-locked to 4 Hz oscillations increased after learning, suggesting more efficient communication between the pStr and visual processing areas during task execution. Notably, the elevation of 4 Hz power prior to and at the onset of the judgment epoch, followed by its gradual decline around the typical decision time, suggests that 4 Hz oscillations provide a prospective network-level timing signal that coordinates information flow during the initiation of the judgment process. Additionally, we found no significant difference in 4 Hz coherence between success and failure trials, even in the early stages when the failure rates were relatively high. This suggests that, unlike midfrontal theta oscillations, which often increase around errors and conflict,^72^^,^^73^ the 4 Hz coherence between the pStr and visual areas primarily reflects learning-dependent changes. Although not statistically significant, we also noted a trend toward decreased theta power in the VC across training. This may reflect reduced attentional demand as the task became more automatic, consistent with reports that theta power often diminishes once behaviors are well learned.^74^^,^^75^^,^^76^

Furthermore, the timing of neuronal spikes to a specific oscillation is also important for integrating external inputs with the internal network dynamics.^32^ We found that the preferred phase of 4 Hz oscillations differed across regions, suggesting a sequence of activation events across the pStr neuronal populations. Neurons phase-locked to the dLGN and VC 4 Hz oscillations typically fired before the peak of the oscillation (∼300°), potentially contributing to the transmission of visual information, whereas those phase-locked to the local pStr oscillation tended to fire near the trough (∼180°), which may reflect integration or output of the task-related information at the subsequent phase. Such phase offsets may facilitate efficient inter-regional information transfer by temporally separating input from integration and output. Although pStr neurons exhibited a degree of position selectivity across the judgment zone, this selectivity did not significantly correlate with their 4 Hz phase-locking, suggesting that spatial tuning and rhythmic synchronization may represent largely independent dimensions of pStr activity. These findings indicate that 4 Hz oscillations in the pStr, potentially driven by the dopaminergic system, facilitate functional connectivity with the visual areas, thereby optimizing the integration of sensory information required for accurate visual discrimination.

In summary, we demonstrated that learning a visual discrimination task is associated with increased pStr activity, enhanced 4 Hz coherence with visual processing areas, and greater phase-locking of the pStr neurons to oscillatory activity. These findings provide insight into how striatal circuits interact with sensory systems to support goal-directed behavior over extended learning periods. Notably, 4 Hz synchronization has been extensively studied in the prefrontal circuits, but our study sheds light on its role in more general sensory processing.

### Limitations of the study

Although our findings emphasize the role of the pStr in visual discrimination learning and its interaction with visual processing regions, several limitations should be considered. First, the current study is correlational, and although increased RS neuronal activity and 4 Hz synchronization were observed in the late learning stage, our data have not yet established a causal relationship between these neuronal changes and improved task performance. Anatomical evidence suggests a direct projection from the dLGN to the pStr, but our data do not confirm that learning specifically enhances this direct pathway. The observed 4 Hz coherence could alternatively arise from a common upstream driver or through indirect multi-synaptic routes. Because 4 Hz oscillation and subsequent synchronization emerge intrinsically in nature, it is technically difficult to manipulate the oscillation with current technology.

Second, we used only black and white stimuli, in other words, global luminance stimuli. In this design, the sensory features and associated values were not orthogonalized, posing a challenge in disentangling whether neuronal responses reflect sensitivity to specific sensory features or to their associated values. Although the freely moving preparation is advantageous for observing oscillatory dynamics, it proved difficult for rats to discriminate more complex visual stimuli such as patterns or gratings while running through the judgment zone. Whether our findings generalize to more complex and demanding perceptual learning scenarios remains to be determined; however, our design allowed the systematic tracking of neuronal changes in the same animals throughout the learning stages.

Third, our electrophysiological recordings were restricted to the pStr, dLGN, and VC, guided by anatomical evidence for direct projections and our focus on cortico-thalamo-striatal communication during visual discrimination. While hippocampal activity is well known for its strong theta oscillations in navigation and memory tasks, previous studies have shown that neurons in the VC also exhibit spatial representations^77^^,^^78^ and theta-band modulations.^79^ In addition, our task relied on continuously available visual cues, making it largely independent of working memory or hippocampal-dependent mechanisms.

Despite these limitations, our findings that the pStr synchronizes with visual areas at 4 Hz after learning offer a novel entry point for exploring the functional significance of low-frequency oscillations in visual cognitive processing. This work lays the groundwork for future investigations into how oscillatory dynamics support learning and sensory integration within distributed neuronal circuits.

## Resource availability

### Lead contact

Requests for further information and resources should be directed to and will be fulfilled by the lead contact, Shigeyoshi Fujisawa (shigeyoshi.fujisawa@riken.jp).

### Materials availability

This study did not generate new unique reagents.

### Data and code availability

• The data described in this article have been deposited at the RIKEN CBS Data Sharing Platform and are publicly available at https://doi.org/10.60178/cbs.20251009-001 as of the date of publication.
• All original code has been deposited at the RIKEN CBS Data Sharing Platform and is publicly available at https://doi.org/10.60178/cbs.20251009-001 as of the date of publication.
• Any additional information required to reanalyze the data reported in this article is available from the lead contact upon request.

## Acknowledgments

We thank the RIKEN Advanced Manufacturing Support Team for support in fabricating the custom-made experimental apparatus, the RIKEN CBS Research Resources Division for support in animal care and the production of custom-made viral vectors. We also thank S. Kashiwagi for support in histological procedures, Y. Sekine for support in animal training, and M. Kondo for helpful discussions. This work was supported by 10.13039/501100001691JSPS 10.13039/501100001691KAKENHI grants (23H02595 for S.F., 22J13309 and 24K23252 for S.T.), 10.13039/100008732Uehara Memorial Foundation (S.F.), and RIKEN 10.13039/100015103JRA fellowship (S.T.).

## Author contributions

S.T. and S.F. designed the experiments. S.T. performed the experiments and analyzed the data. S.T. and S.F. wrote the article.

## Declaration of interests

The authors declare no competing interests.

## STAR★Methods

### Key resources table

**Table:** 

REAGENT or RESOURCE	SOURCE	IDENTIFIER
**Antibodies**		

Rabbit anti-dsRed	Takara Bio	Cat# 632496; RRID:AB_10013483
Chicken anti-GFP	Antibodies Incorporated	Cat# GFP-1010; RRID:AB_2307313
Donkey anti-Rabbit Alexa 546	Jackson ImmunoResearch Labs	Cat# 703-545-155; RRID:AB_2340375
Donkey anti-Chicken Alexa 488	Thermo Fisher Scientific	Cat# A10040; RRID:AB_2534016

**Bacterial and virus strains**		

AAV1-Ef1a-fDIO-tdTomato	Addgene	Cat #128434
AAV2-hSyn-DIO-EGFP	Addgene	Cat #50457
AAV1-hSyn-Cre-WPRE-hGH	Addgene	Cat #105553
pAdDeltaF6	Addgene	Cat #112867
pAAV2/1	Addgene	Cat #112862
pAAV phSyn1(S)-FlpO-bGHpA	Addgene	RRID:Addgene_51669

**Biological samples**		

293T	RIKEN BRC	N/A

**Deposited data**		

Behavioral and electrophysiological data	This study	https://doi.org/10.60178/cbs.20251009-001

**Experimental models: Organisms/strains**		

Rat (Long-Evans)	Japan SLC	Cat# Iar:Long-Evans

**Software and algorithms**		

MATLAB	MathWorks	https://ww2.mathworks.cn/products/matlab.html; RRID:SCR_001622
LabVIEW	National Instruments	RRID:SCR_014325
Python	https://www.python.org	RRID:SCR_008394
Fiji (ImageJ)	NIH	https://imagej.net/; RRID:SCR_003070
Kilosort	https://github.com/cortex-lab/Kilosort	RRID:SCR_016422
Phy	https://github.com/cortex-lab/phy	N/A
DeepLabCut	https://github.com/DeepLabCut/DeepLabCut	RRID:SCR_021391
Analysis code	This study	https://doi.org/10.60178/cbs.20251009-001

**Other**		

Silicon probe: 64-sites: 6-shank probe	NeuroNexus	Buzsaki64sp, Buzsaki64spL
Silicon probe: 64-sites: 5-shank probe	NeuroNexus	Buzsaki5x12
Silicon probe: 128-sites: 8-shank probe	Diagnostic Biochips	P128-8
1024-channel RHD Recording System	Intan Technologies	RRID: SCR_019278

### Experimental model and study participant details

All animal experiments were approved by the RIKEN Institutional Animal Care and Use Committee. Male Long-Evans rats (aged 7 to 8 weeks at the start of the behavioral training; SLC, Hamamatsu, Shizuoka, Japan) were used for the experiments in this study. The rats had not been used in any other experiments prior to this study. All rats were provided food and water *ad libitum* before the behavioral training sessions, and were housed in a temperature-controlled room (20° to 22°C) under a light-dark cycle (lights on from 8:00 to 20:00). All behavioral sessions were conducted during the light period.

### Method details

#### Behavioral training

Rats were water-deprived in their home cages when they weighed more than 210 g. They received approximately 4-8 mL of water per session each day, and were sometimes given additional water to maintain their body weight at more than 90% of their initial weight throughout the experiments. The rats were usually trained for six consecutive days per week, and received a 10 g agar block (Oriental Yeast Co., Ltd., Tokyo, Japan) per day without training.

The training apparatus was a modified figure-8 T-maze (Figure 1A) with a wide central arm where the sample stimuli (black or white) were presented on the monitors on both sides. The arms were positioned 52 cm above the room floor and separated by automatic doors (W35 cm by H40 cm; IABL3826, IAI Corporation, Shizuoka, Japan). Two water ports (D2 cm by H1.5 cm) for reward were positioned at the ends of the T-shaped arms, and the home zone also had another water port. These water ports were connected to micropumps (Type 7615; Christian Bürkert GmbH & Co. KG, Ingelfingen, Germany) that delivered 0.2% saccharin water (30432-62; NACALAI TESQUE, INC., Kyoto, Japan) as a reward. For system control, five infrared sensors (PR-M51CP, PR-F51CP; Keyence, Osaka, Japan) were placed in the apparatus to detect the rats’ positions (Figure 1A). The rats’ positions were also monitored with a CMOS camera (acA720-290gc, Basler AG, Ahrensburg, Germany) with a wide-angle lens (TF2.8DA-8; FUJIFILM Co., Tokyo, Japan) set on the ceiling.

Behavioral experiments were automatically controlled by custom-written LabVIEW (National Instruments, Austin, TX) programs and the images from the CMOS camera were acquired at 10-30 Hz using pylon7 Viewer (Basler AG, Ahrensburg, Germany) on a Windows PC.

##### Pre-training

On the first day of pre-training, rats were placed in the T-maze apparatus without doors and allowed to explore freely for approximately 40 min. From the second day onwards, the rats were trained to run through the apparatus with doors that automatically opened and closed. In this training session, a trial was initiated by opening a door at the entrance to the judgment zone, and the rats ran through either side of the arms with door open. The rats were given approximately 60 μL of saccharine water at the end of the arm. After collecting the reward water, rats came back to the home zone where approximately 10 μL of saccharine water was delivered. After 4-7 days of training, the rats were able to complete more than 60 trials per session within an hour and then they began the visual discrimination task.

##### Visual discrimination task

Rats were allowed to initiate a trial by crossing a sensor at the entrance of the central arm (judgment zone; Figure 1A) with their timing after the entrance door opened. The monitors on both sides of the judgment zone were set to either white (Stim 1) or black (Stim 2) before the door opened. Rats were required to choose the left or right arm depending on the color of the monitors, where the monitor color defined the global luminance level of the judgment zone. If the monitors were both white (Stim 1), the rats received 60 μL of saccharine water at the end of the left arm and nothing when they chose the right arm. If the monitors were both black (Stim 2), they received reward water on the right arm and nothing on the left arm (Figure 1B). Both monitors turned to gray when the rats exited the judgment zone.

When the rats began to choose both arms almost equally, they underwent electrode implantation surgery. After recovery from surgery, they were water-deprived again, went through several pre-training sessions to habituate the recording cables and then trained this visual discrimination task with electrophysiological recording for about 1-2 months.

#### Surgery

##### Silicon probe implantation

Rats were anesthetized with isoflurane (5% for induction and 1.8-2.3% during surgery; Pfizer Japan, Tokyo, Japan) inhalation, treated with an eye ointment (Neo-Medrol EE; Pfizer Japan, Tokyo, Japan), and placed on a stereotaxic frame (Model 962; Kopf, Tujunga, CA). Before the incision, hairs in the operative field were removed and sterilized with 10% isodine solution (Mundipharma, Tokyo, Japan).

Craniotomies over either left or right pStr (3.1 mm posterior to bregma, 4.7 – 6.3 mm lateral from the midline), left dLGN (4.5 mm posterior to bregma, 2.5 – 4.1 mm lateral from the midline), and left VC (6.2 – 7.8 mm posterior to bregma, 4.5 or 6 mm lateral from the midline) were performed using dental drills (NSK Volvere Vmax; Nakanishi Inc., Tochigi, Japan; Komet HP Carbide burs #1/2; Momose Dental MFG CO., LTD, Osaka, Japan) (Figure S3). In this study, we used three types of silicon probes: Buzsaki 64sp/64spL (NeuroNexus, Ann Arbor, MI), which consisted of six shanks (200 μm shank separation) and 10 recording sites for each shank (20 μm vertical separation; 160 μm^2^ each site; ∼1 MΩ impedance), Buzsaki 5x12 (NeuroNexus, Ann Arbor, MI), which consisted of five shanks (200 μm shank separation) and 12 recording sites for each shank (20 μm vertical separation; 160 μm^2^ each site; ∼1 MΩ impedance), and P128-8 (Diagnostic Biochips, Glen Burine, MD), which consisted of eight shanks (150 μm shank separation) and 16 recording sites for each shank (30 μm vertical and 16.5 μm lateral separation; 165 μm^2^ each site; ∼1 MΩ impedance). Either Buzsaki 64spL or P128-8 was implanted in the pStr, Buzsaki 64spL was implanted in the dLGN and either Buzsaki 64sp or Buzsaki 5x12 was implanted in the VC. The silicon probes were attached to a micromanipulator and gradually moved to the desired depth position (pStr and dLGN: 4 mm, VC: 0.8 mm from the brain surface). The brain surface was protected with silicone oil (Sigma-Aldrich, St. Louis, MO) and the probes were covered with paraffin (NACALAI TESQUE, INC., Kyoto, Japan). The detailed surgical procedures are described elsewhere.^53^^,^^80^

After the implantation surgery, the silicon probes were lowered every day until the estimated depth reached the target position. The rats were allowed to recover for more than 5 days before behavioral training so that they gained 20 g more weight than before surgery.

During the recording sessions, the wide-band neurophysiological signals were recorded continuously at 20 kHz using the Intan RHD 1024-channel recording controller (Intan Technologies, Los Angeles, CA). Signals down-sampled to 1.25 kHz were used for LFP analysis.

##### Virus injection

Rats were anesthetized with isoflurane inhalation and the same preparation as for the electrode implantation was made except that craniotomies were performed over the pStr (3.1 mm posterior to bregma, 5.5 mm lateral from the midline), dLGN (4.5 mm posterior to bregma, 3.7 mm lateral from the midline), and VC (7 mm posterior to bregma, 4.5 and 5.5 mm lateral from the midline). All the injections were performed using a glass pipette connected by silicone tubing to a 50 μL syringe (Hamilton, Reno, NV) at the rate of 30-50 nL/min. 300 nL of 1:1 solution of AAV1-Ef1a-fDIO-tdTomato and AAV2-hSyn-DIO-EGFP was injected into the pStr (4.2 mm and 4.5 mm from the brain surface), 300 nL of AAV1-hSyn-Cre-WPRE-hGH was injected into the dLGN (4.3 mm from the brain surface) and 500 nL of AAV1-phSyn1(S)-FlpO-bGHpA was injected into the VC (1.2 mm from the brain surface). AAV1-phSyn1(S)-FlpO-bGHpA was produced in RIKEN BMA using plasmid obtained from Addgene (Watertown, MA). 293T was provided by the RIKEN BRC through National BioResource Project of the MEXT/AMED, Japan. All the other viruses were purchased from Addgene. The pipette was left in place for 10 minutes after the injections and then slowly withdrawn. The craniotomy was covered with silicone elastomer (Kwik-Cast, World Precision Instruments, FL) and the incision was closed with 5-0 nylon sutures (Natsume Seisakusho Co., Tokyo, Japan). The rats were returned to their home cages and remained there for 7-8 weeks before perfusion.

#### Histology

Rats were deeply anesthetized with 5% isoflurane inhalation and transcardially perfused with 200-300 mL of phosphate buffered saline (PBS) and the same volume of 4% paraformaldehyde (PFA) in PBS as previously described.^33^ Brains were removed, postfixed in PFA solution at 4°C for at least 12 hours and then sliced into 100-μm sections.

##### Localization of recording sites

To identify the depth location of a specific recording site, a small current (1 μA for 10-15 s) was passed through the platinum-iridium recording pad of the probe two days prior to perfusion. The sliced sections were mounted on slides with DAPI mounting medium (DAPI Fluoromount-G; SouthernBiotech, Birmingham, AL).

##### Immunostaining

For neural tracing, sections were rinsed in PBS-X (2% Triton-X (NACALAI TESQUE, INC., Kyoto, Japan) in PBS) containing 5% normal donkey serum (D9663; Sigma-Aldrich, St. Louis, MO) and incubated with the primary antibodies (1:867 dilution of rabbit anti-dsRed (632496; Takara Bio Inc., Shiga, Japan) and 1:464 dilution of chicken anti-GFP (GFP-1010; Antibodies Incorporated, Davis, CA)) for more than 12 hours at 4°C. They were then rinsed in PBS-X and incubated with species-appropriate Alexa Fluor 488 (703-545-155; Jackson ImmunoResearch, Cambridgeshire, United Kingdom) and 546 (A10040; Thermo Fisher Scientific Inc., Waltham, MA) conjugated secondary antibodies at the same dilution as the primary antibodies. The sections were mounted on slides with DAPI mounting medium (DAPI Fluoromount-G; SouthernBiotech, Birmingham, AL).

Fluorescence images were acquired using virtual slide scanners (NDP; Hamamatsu Photonics, Shizuoka, Japan or VS200; Olympus, Tokyo, Japan) or an all-in-one fluorescence microscope (BZ-X700, Keyence, Osaka, Japan). Detailed images were captured with a confocal microscope (FV3000; Olympus, Tokyo, Japan) and analyzed using ImageJ Fiji software.^81^^,^^82^

#### Data analysis

##### Behavioral data analysis

We used the success rate and reaction time to estimate the learning phase. Reaction time was calculated as the latency from judgment-zone entry to arrival at the reward port. To determine the learning stages, hierarchical clustering was applied using ‘clustergram.m’ function with Ward’s method in MATLAB Bioinformatics Toolbox.

The head positions of rats during the visual discrimination task were detected and tracked using DeepLabCut.^41^ Judgment distance was defined as the distance from the entrance of the judgment zone to the point where the rats’ trajectories changed significantly between left- and right-chosen trials per session. We estimated the significant point using the permutation methods (see below).^53^ Judgment time was defined as the time it took the rats to run the judgment distance. The instantaneous running speed of the rats was calculated from the travel distance at each time step and subsequently smoothed using a Gaussian filter with a sigma of 2.

##### Spike detection

Spike sorting was performed semi-automatically with Kilosort 2.0^83^ followed by the manual curation with Phy (https://github.com/cortex-lab/phy).

##### Spike data analysis

All the curated units were classified as RS (regular-spiking) or FS (fast-spiking) neurons based on their spike width and firing rate.^55^^,^^56^^,^^57^^,^^58^ Spike width was calculated based on the duration during which the amplitude was less than one third of the trough-to-peak amplitude (Figure 2B). PROP (ISI > 2 s) was defined by the proportion of time associated with long (> 2 s) inter-spike-intervals (ISIs), summing ISIs longer than 2 s and dividing the sum by the total recording time (Figure S4A). Given the considerable inter-animal variability in time taken to traverse the judgment zone (coefficient of variation = 17.7%), we evaluated the firing rates at each position within the judgment zone. To this end, we introduced a normalized distance metric, defined such that the start of the judgment zone was zero (a sensor there controlled the start of trials) and the end of the judgment zone was one (a sensor there controlled the monitor to turn gray). Firing rates were normalized by the amount of time the rats spent at each position. The positions where the firing rates were significantly different between trials presented with Stim 1 and 2 were determined based on the permutation test (*p* < 0.05).^53^ We used mutual information to calculate spatial information (bit/spike).^54^

##### LFP data analysis

Wavelet transformation and wavelet coherence analysis were applied to raw LFPs in the pStr, dLGN, and VC, using ‘cwt.m’ with Morlet wavelet and ‘wcoherence.m’ functions in MATLAB Wavelet Toolbox. For 4 Hz and theta phase extraction, LFPs in the pStr, dLGN, and VC were filtered with a Butterworth filter with a passband range of 2-5 Hz and 6-10 Hz, respectively. Instantaneous 4 Hz and theta phases were estimated by Hilbert transformation of the filtered signals.

##### Phase modulation analysis

The Rayleigh test was used to assess the uniformity of the spike histogram based on the 4 Hz oscillation phase. The statistics Z = R^2^/n (R: resultant length, n: number of samples), or variance-stabilized log(Z) was used for significance test.^33^^,^^84^

##### Permutation test

Permutation tests were used to identify conditional differences in rat trajectories or firing rates. Detailed information of this method is described in previous papers.^53^^,^^85^ Briefly, differences in mean left/right trajectories or Stim 1/2 firing rates were calculated. Then, the assignments of each condition to the trials were randomly permuted and the differences of the averaged trajectories or firing rates based on the permuted labels were re-estimated 5000 times. Using this shuffled dataset, a two-sided pointwise p-value (α = 0.05) was computed at each point. Finally, to avoid multiple comparison issues, the global band level was determined by calculating the percentage of datasets that exceeded each pointwise band level, and the value corresponding to the 5% bands were computed.^53^

### Quantification and statistical analysis

All the data were analyzed using MATLAB R2019b or R2021a (MathWorks, Natick, MA). The statistical details are provided in the results section and the figure legends. All statistical tests were two-tailed. For multiple comparisons, we first performed one- or two-way ANOVA to identify pairs exhibiting significant differences. Post hoc Wilcoxon signed rank test or Wilcoxon rank-sum test was then applied to these pairs to confirm the reliability of the observed differences. Permutation tests were applied to identify significant points between the two curves. The Rayleigh test was used to assess circular uniformity. The Kolmogorov-Smirnov test was applied to compare cumulative distributions (‘kstest.m’ function in MATLAB Statistics and Machine Learning Toolbox). Clopper-Pearson intervals were used to calculate binominal confidence intervals^86^ (‘binofit.m’ function in MATLAB Statistics and Machine Learning Toolbox). Data are presented as mean ± standard error of the mean unless otherwise noted.

## References

[1] Znamenskiy P., Zador A.M. (2013). Corticostriatal neurons in auditory cortex drive decisions during auditory discrimination. Nature.

[2] Xiong Q., Znamenskiy P., Zador A.M. (2015). Selective corticostriatal plasticity during acquisition of an auditory discrimination task. Nature.

[3] Guo L., Weems J.T., Walker W.I., Levichev A., Jaramillo S. (2019). Choice-Selective Neurons in the Auditory Cortex and in Its Striatal Target Encode Reward Expectation. J. Neurosci..

[4] Nardoci M.B., Lakunina A.A., Henderling D.C., Pedregon J.C., Mohn J.L., Jaramillo S. (2022). Sound-evoked responses of distinct neuron classes from the tail of the striatum. eNeuro.

[5] Cui L., Tang S., Pan J., Deng L., Zhang Z., Zhao K., Si B., Xu N.L. (2025). Causal contributions of cell-type-specific circuits in the posterior dorsal striatum to auditory decision-making. Cell Rep..

[6] Menegas W., Bergan J.F., Ogawa S.K., Isogai Y., Umadevi Venkataraju K., Osten P., Uchida N., Watabe-Uchida M. (2015). Dopamine neurons projecting to the posterior striatum form an anatomically distinct subclass. eLife.

[7] Hunnicutt B.J., Jongbloets B.C., Birdsong W.T., Gertz K.J., Zhong H., Mao T. (2016). A comprehensive excitatory input map of the striatum reveals novel functional organization. eLife.

[8] Hintiryan H., Foster N.N., Bowman I., Bay M., Song M.Y., Gou L., Yamashita S., Bienkowski M.S., Zingg B., Zhu M. (2016). The mouse cortico-striatal projectome. Nat. Neurosci..

[9] Menegas W., Babayan B.M., Uchida N., Watabe-Uchida M. (2017). Opposite initialization to novel cues in dopamine signaling in ventral and posterior striatum in mice. eLife.

[10] Jiang H., Kim H.F. (2018). Anatomical Inputs From the Sensory and Value Structures to the Tail of the Rat Striatum. Front. Neuroanat..

[11] Lee K., An S.-Y., Park J., Lee S., Kim H.F. (2023). Anatomical and functional comparison of the caudate tail in primates and the tail of the striatum in rodents: Implications for sensory information processing and habitual behavior. Mol. Cells.

[12] Brown V.J., Desimone R., Mishkin M. (1995). Responses of cells in the tail of the caudate nucleus during visual discrimination learning. J. Neurophysiol..

[13] Yamamoto S., Kim H.F., Hikosaka O. (2013). Reward Value-Contingent Changes of Visual Responses in the Primate Caudate Tail Associated with a Visuomotor Skill. J. Neurosci..

[14] Kim H.F., Hikosaka O. (2013). Distinct Basal Ganglia Circuits Controlling Behaviors Guided by Flexible and Stable Values. Neuron.

[15] Hikosaka O., Yamamoto S., Yasuda M., Kim H.F. (2013). Why skill matters. Trends Cogn. Sci..

[16] Ding L., Gold J.I. (2013). The basal ganglia’s contributions to perceptual decision making. Neuron.

[17] Seger C.A. (2013). The visual corticostriatal loop through the tail of the caudate: circuitry and function. Front. Syst. Neurosci..

[18] Anderson B.A., Laurent P.A., Yantis S. (2014). Value-driven attentional priority signals in human basal ganglia and visual cortex. Brain Res..

[19] Hikosaka O., Kim H.F., Yasuda M., Yamamoto S. (2014). Basal ganglia circuits for reward value-guided behavior. Annu. Rev. Neurosci..

[20] Amita H., Hikosaka O. (2019). Indirect pathway from caudate tail mediates rejection of bad objects in periphery. Sci. Adv..

[21] Amita H., Kim H.F., Smith M.K., Gopal A., Hikosaka O. (2019). Neuronal connections of direct and indirect pathways for stable value memory in caudal basal ganglia. Eur. J. Neurosci..

[22] Amita H., Kim H.F., Inoue K.-I., Takada M., Hikosaka O. (2020). Optogenetic manipulation of a value-coding pathway from the primate caudate tail facilitates saccadic gaze shift. Nat. Commun..

[23] Choi Y., Shin E.Y., Kim S. (2020). Spatiotemporal dissociation of fMRI activity in the caudate nucleus underlies human *de novo* motor skill learning. Proc. Natl. Acad. Sci. USA.

[24] Kang J., Kim H., Hwang S.H., Han M., Lee S.H., Kim H.F. (2021). Primate ventral striatum maintains neural representations of the value of previously rewarded objects for habitual seeking. Nat. Commun..

[25] Kunimatsu J., Amita H., Hikosaka O. (2024). Neuronal response of the primate striatum tail to face of socially familiar persons. iScience.

[26] Kintscher M., Kochubey O., Schneggenburger R. (2023). A striatal circuit balances learned fear in the presence and absence of sensory cues. eLife.

[27] Druart M., Kori M., Chaimowitz C., Fan C., Sippy T. (2025). Cell-type-specific auditory responses in the striatum are shaped by feedforward inhibition. Cell Rep..

[28] Sherman S.M., Guillery R.W. (2002). The role of the thalamus in the flow of information to the cortex. Philos Trans R Soc Lond B Biol Sci..

[29] Saalmann Y.B., Kastner S. (2011). Cognitive and Perceptual Functions of the Visual Thalamus. Neuron.

[30] Einstein M.C., Polack P.-O., Tran D.T., Golshani P. (2017). Visually evoked 3-5 Hz membrane potential oscillations reduce the responsiveness of visual cortex neurons in awake behaving mice. J. Neurosci..

[31] Shin D., Peelman K., Lien A.D., Del Rosario J., Haider B. (2023). Narrowband gamma oscillations propagate and synchronize throughout the mouse thalamocortical visual system. Neuron.

[32] Buzsáki G., Draguhn A. (2004). Neuronal Oscillations in Cortical Networks. Science.

[33] Fujisawa S., Buzsáki G. (2011). A 4 Hz Oscillation Adaptively Synchronizes Prefrontal, VTA, and Hippocampal Activities. Neuron.

[34] Parker K.L., Chen K.-H., Kingyon J.R., Cavanagh J.F., Narayanan N.S. (2014). D1-dependent 4 Hz oscillations and ramping activity in rodent medial frontal cortex during interval timing. J. Neurosci..

[35] Emmons E.B., Ruggiero R.N., Kelley R.M., Parker K.L., Narayanan N.S. (2016). Corticostriatal field potentials are modulated at delta and theta frequencies during interval-timing task in rodents. Front. Psychol..

[36] Duvarci S., Simpson E.H., Schneider G., Kandel E.R., Roeper J., Sigurdsson T. (2018). Impaired recruitment of dopamine neurons during working memory in mice with striatal D2 receptor overexpression. Nat. Commun..

[37] Clarke-Williams C.J., Lopes-Dos-Santos V., Lefèvre L., Brizee D., Causse A.A., Rothaermel R., Hartwich K., Perestenko P.V., Toth R., McNamara C.G. (2024). Coordinating brain-distributed network activities in memory resistant to extinction. Cell.

[38] Parker K.L., Chen K.-H., Kingyon J.R., Cavanagh J.F., Narayanan N.S. (2015). Medial frontal ∼4-Hz activity in humans and rodents is attenuated in PD patients and in rodents with cortical dopamine depletion. J. Neurophysiol..

[39] Halje P., Brys I., Mariman J.J., da Cunha C., Fuentes R., Petersson P. (2019). Oscillations in cortico-basal ganglia circuits: implications for Parkinson’s disease and other neurologic and psychiatric conditions. J. Neurophysiol..

[40] Singh A., Cole R.C., Espinoza A.I., Evans A., Cao S., Cavanagh J.F., Narayanan N.S. (2021). Timing variability and midfrontal ∼4 Hz rhythms correlate with cognition in Parkinson’s disease. npj Parkinson's Dis..

[41] Nath T., Mathis A., Chen A.C., Patel A., Bethge M., Mathis M.W. (2019). Using DeepLabCut for 3D markerless pose estimation across species and behaviors. Nat. Protoc..

[42] Mallet N., Le Moine C., Charpier S., Gonon F. (2005). Feedforward inhibition of projection neurons by fast-spiking GABA interneurons in the rat striatum *in vivo*. J. Neurosci..

[43] Pimentel-Farfan A.K., Báez-Cordero A.S., Peña-Rangel T.M., Rueda-Orozco P.E. (2022). Cortico-striatal circuits for bilaterally coordinated movements. Sci. Adv..

[44] Rios A., Nonomura S., Kato S., Yoshida J., Matsushita N., Nambu A., Takada M., Hira R., Kobayashi K., Sakai Y. (2023). Reward expectation enhances action-related activity of nigral dopaminergic and two striatal output pathways. Commun. Biol..

[45] Berke J.D., Okatan M., Skurski J., Eichenbaum H.B. (2004). Oscillatory entrainment of striatal neurons in freely moving rats. Neuron.

[46] Yamin H.G., Stern E.A., Cohen D. (2013). Parallel processing of environmental recognition and locomotion in the mouse striatum. J. Neurosci..

[47] Barter J.W., Li S., Lu D., Bartholomew R.A., Rossi M.A., Shoemaker C.T., Salas-Meza D., Gaidis E., Yin H.H. (2015). Beyond reward prediction errors: the role of dopamine in movement kinematics. Front. Integr. Neurosci..

[48] Peters A.J., Fabre J.M.J., Steinmetz N.A., Harris K.D., Carandini M. (2021). Striatal activity topographically reflects cortical activity. Nature.

[49] Miyamoto Y., Nagayoshi I., Nishi A., Fukuda T. (2019). Three divisions of the mouse caudal striatum differ in the proportions of dopamine D1 and D2 receptor-expressing cells, distribution of dopaminergic axons, and composition of cholinergic and GABAergic interneurons. Brain Struct. Funct..

[50] Valjent E., Gangarossa G. (2021). The Tail of the Striatum: From Anatomy to Connectivity and Function. Trends Neurosci..

[51] Gage G.J., Stoetzner C.R., Wiltschko A.B., Berke J.D. (2010). Selective activation of striatal fast-spiking interneurons during choice execution. Neuron.

[52] Yarom O., Cohen D. (2011). Putative cholinergic interneurons in the ventral and dorsal regions of the striatum have distinct roles in a two choice alternative association task. Front. Syst. Neurosci..

[53] Fujisawa S., Amarasingham A., Harrison M.T., Buzsáki G. (2008). Behavior-dependent short-term assembly dynamics in the medial prefrontal cortex. Nat. Neurosci..

[54] Skaggs W., McNaughton B., Gothard K. (1992). An information-theoretic approach to deciphering the hippocampal code. Adv. Neural Inf. Process. Syst..

[55] Niell C.M., Stryker M.P. (2008). Highly selective receptive fields in mouse visual cortex. J. Neurosci..

[56] Isomura Y., Harukuni R., Takekawa T., Aizawa H., Fukai T. (2009). Microcircuitry coordination of cortical motor information in self-initiation of voluntary movements. Nat. Neurosci..

[57] Durand S., Iyer R., Mizuseki K., de Vries S., Mihalas S., Reid R.C. (2016). A comparison of visual response properties in the lateral geniculate nucleus and primary visual cortex of awake and anesthetized mice. J. Neurosci..

[58] Kirchgessner M.A., Franklin A.D., Callaway E.M. (2020). Context-dependent and dynamic functional influence of corticothalamic pathways to first- and higher-order visual thalamus. Proc. Natl. Acad. Sci. USA.

[59] Evangelio M., García-Amado M., Clascá F. (2018). Thalamocortical projection neuron and interneuron numbers in the visual thalamic nuclei of the adult C57BL/6 mouse. Front. Neuroanat..

[60] Wang L., Rangarajan K.V., Gerfen C.R., Krauzlis R.J. (2018). Activation of Striatal Neurons Causes a Perceptual Decision Bias during Visual Change Detection in Mice. Neuron.

[61] Cox J., Witten I.B. (2019). Striatal circuits for reward learning and decision-making. Nat. Rev. Neurosci..

[62] Chen A.P.F., Malgady J.M., Chen L., Shi K.W., Cheng E., Plotkin J.L., Ge S., Xiong Q. (2022). Nigrostriatal dopamine pathway regulates auditory discrimination behavior. Nat. Commun..

[63] Cover K.K., Elliott K., Preuss S.M., Krauzlis R.J. (2025). A distinct circuit for biasing visual perceptual decisions and modulating superior colliculus activity through the mouse posterior striatum. bioRxiv.

[64] Kawaguchi Y., Wilson C.J., Augood S.J., Emson P.C. (1995). Striatal interneurones: chemical, physiological and morphological characterization. Trends Neurosci..

[65] Silberberg G., Bolam J.P. (2015). Local and afferent synaptic pathways in the striatal microcircuitry. Curr. Opin. Neurobiol..

[66] Ogata K., Kadono F., Hirai Y., Inoue K.I., Takada M., Karube F., Fujiyama F. (2022). Conservation of the Direct and Indirect Pathway Dichotomy in Mouse Caudal Striatum With Uneven Distribution of Dopamine Receptor D1- and D2-Expressing Neurons. Front. Neuroanat..

[67] Berke J.D. (2011). Functional properties of striatal fast-spiking interneurons. Front. Syst. Neurosci..

[68] Kumar A., Guo L. (2024). Functional consequences of fast-spiking interneurons in striatum. bioRxiv.

[69] Kim Y.-C., Han S.-W., Alberico S.L., Ruggiero R.N., De Corte B., Chen K.-H., Narayanan N.S. (2017). Optogenetic stimulation of frontal D1 neurons compensates for impaired temporal control of action in dopamine-depleted mice. Curr. Biol..

[70] Scangos K.W., Carter C.S., Gurkoff G., Zhang L., Shahlaie K. (2018). A pilot study of subthalamic theta frequency deep brain stimulation for cognitive dysfunction in Parkinson’s disease. Brain Stimul..

[71] Salehi N., Nahrgang S., Petershagen W., Dembek T.A., Pedrosa D., Timmermann L., Weber I., Oehrn C.R. (2024). Theta frequency deep brain stimulation in the subthalamic nucleus improves working memory in Parkinson’s disease. Brain.

[72] Cohen M.X., Elger C.E., Fell J. (2009). Oscillatory activity and phase-amplitude coupling in the human medial frontal cortex during decision making. J. Cogn. Neurosci..

[73] Cavanagh J.F., Cohen M.X., Allen J.J.B. (2009). Prelude to and resolution of an error: EEG phase synchrony reveals cognitive control dynamics during action monitoring. J. Neurosci..

[74] Clements G.M., Bowie D.C., Gyurkovics M., Low K.A., Fabiani M., Gratton G. (2021). Spontaneous alpha and theta oscillations are related to complementary aspects of cognitive control in younger and older adults. Front. Hum. Neurosci..

[75] Martínez-Molina M.P., Valdebenito-Oyarzo G., Soto-Icaza P., Zamorano F., Figueroa-Vargas A., Carvajal-Paredes P., Stecher X., Salinas C., Valero-Cabré A., Polania R., Billeke P. (2024). Lateral prefrontal theta oscillations causally drive a computational mechanism underlying conflict expectation and adaptation. Nat. Commun..

[76] Scaramuzzi G.F., Spina A.C., Manippa V., Amico F., Cornacchia E., Palmisano A., Scianatico G., Buscombe R., Avery R., Thoma V., Rivolta D. (2025). Darts fast-learning reduces theta power but is not affected by Hf-tRNS: A behavioral and electrophysiological investigation. Brain Res..

[77] Ji D., Wilson M.A. (2007). Coordinated memory replay in the visual cortex and hippocampus during sleep. Nat. Neurosci..

[78] Fournier J., Saleem A.B., Diamanti E.M., Wells M.J., Harris K.D., Carandini M. (2020). Mouse visual cortex is modulated by distance traveled and by theta oscillations. Curr. Biol..

[79] Levy J.M., Zold C.L., Namboodiri V.M.K., Hussain Shuler M.G. (2017). The timing of reward-seeking action tracks visually cued theta oscillations in primary visual cortex. J. Neurosci..

[80] Vandecasteele M., Royer S., Belluscio M., Berényi A., Diba K., Fujisawa S., Grosmark A., Mao D., Mizuseki K., Patel J. (2012). Large-scale recording of neurons by movable silicon probes in behaving rodents. J. Vis. Exp..

[81] Schindelin J., Arganda-Carreras I., Frise E., Kaynig V., Longair M., Pietzsch T., Preibisch S., Rueden C., Saalfeld S., Schmid B. (2012). Fiji: an open-source platform for biological-image analysis. Nat. Methods.

[82] Schneider C.A., Rasband W.S., Eliceiri K.W. (2012). NIH Image to ImageJ: 25 years of image analysis. Nat. Methods.

[83] Pachitariu M., Steinmetz N., Kadir S., Carandini M., Kenneth D H. (2016). Kilosort: realtime spike-sorting for extracellular electrophysiology with hundreds of channels. bioRxiv.

[84] Sirota A., Montgomery S., Fujisawa S., Isomura Y., Zugaro M., Buzsáki G. (2008). Entrainment of Neocortical Neurons and Gamma Oscillations by the Hippocampal Theta Rhythm. Neuron.

[85] Amarasingham A., Harrison M.T., Hatsopoulos N.G., Geman S. (2012). Conditional modeling and the jitter method of spike resampling. J. Neurophysiol..

[86] Clopper C.J., Pearson E.S. (1934). The use of confidence or fiducial limits illustrated in the case of the binomial. Biometrika.

