# Supplementary material for: Learning-dependent 4 Hz synchronization in the posterior striatum, lateral geniculate nucleus, and visual cortex
Source: iScience. 2025 Nov 6;28(12):113958. doi: 10.1016/j.isci.2025.113958 (PMC12670538; doi:10.1016/j.isci.2025.113958)
Supplement: Document S1. Figures S1–S11 and Table S1 [file mmc1.pdf]

**iScience, Volume *28***

## Supplemental information

# Learning-dependent 4 Hz synchronization in the posterior striatum, lateral geniculate nucleus, and visual cortex

**Sai Tanimoto and Shigeyoshi Fujisawa**

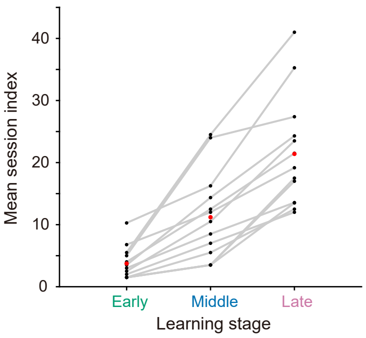


**Figure S1. Mean session index for each learning stage. Related to Figure 1.**
To verify that our stage classification progressed appropriately with learning, we computed the mean session index of the sessions included in each learning stage for each rat. Black dots denote the per-rat means and red dots denote the group average across rats. In every rat, the mean session index increased across progressive learning stages, supporting the validity of the stage classification.

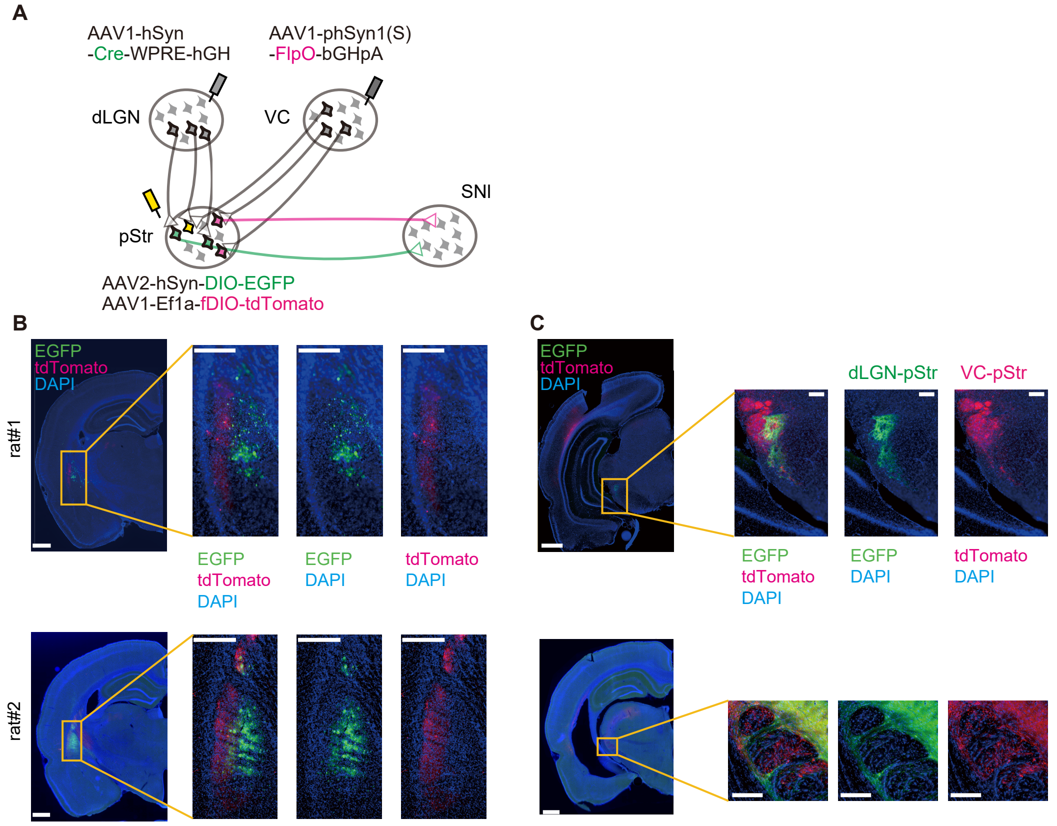


**Figure S2. Innervation of the pStr from both the dLGN and VC. Related to Figure 2.**
(A) Schematic of viral tracing. In order to examine whether the fibers in the pStr from visual areas are passing or projecting fibers, AAV vectors capable of trans-synaptic delivery of Cre or FlpO were injected into the dLGN and VC, respectively. In the pStr, AAV vectors driving EGFP expression in a Cre-dependent manner or tdTomato in a FlpO-dependent manner were injected.
(B) Expression of both EGFP and tdTomato in the pStr cell bodies indicates that the inputs from both the dLGN and VC form true projections rather than fibers of passage. Scale bars: 1 mm for large overview slices at left and 0.5 mm for others.
(C) EGFP- and tdTomato-labeled axon terminals were observed in the substantia nigra pars lateralis (SNl), the principal target of the pStr, further confirming that the pStr is innervated by both the dLGN and VC. Scale bars: 1 mm for large overview slices at left and 0.2 mm for others.

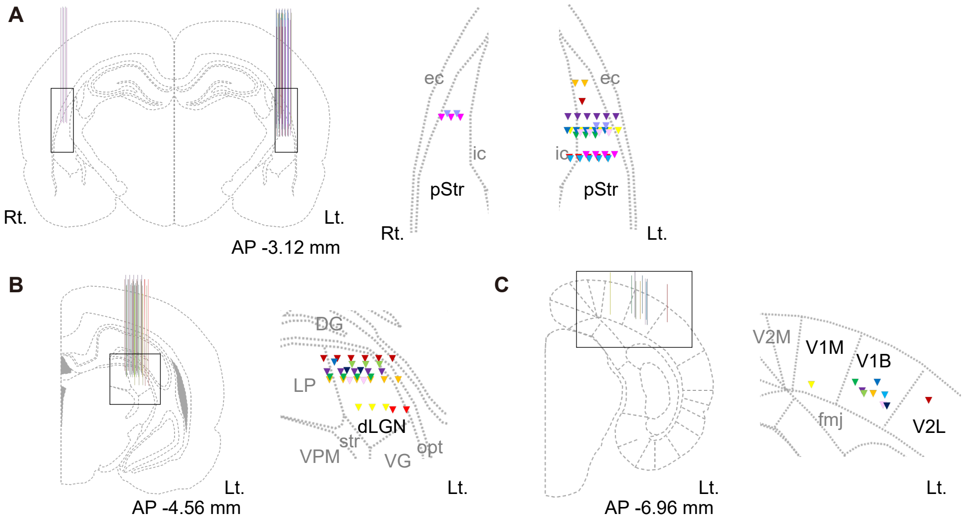


**Figure S3. Summary of electrode tip locations across rats. Related to Figure 2.**
Reconstructed electrode tracks (left) and enlarged views of boxed regions (right) in the pStr (A), dLGN (B), and VC (C) from all rats (N = 11, 10, and 10 for pStr, dLGN, and VC, respectively) are overlaid on corresponding coronal sections from the Paxinos and Watson rat brain atlas[S1]. Each color denotes a different rat. Abbreviations: ec, extrenal capsule; fmj, forceps major of the corpus callosum; ic, internal capsule; opt, optic tract; str, superior thalamic radiation; DG, dentate gyrus; LP, lateral posterior thalamic nucleus; V1B, primary visual cortex binocular area; V1M, primary visual cortex monocular area; V2L, secondary visual cortex lateral area; V2M, secondary visual cortex medial area; VG, ventral geniculate nucleus; VPM, ventral posteromedial thalamic nucleus.

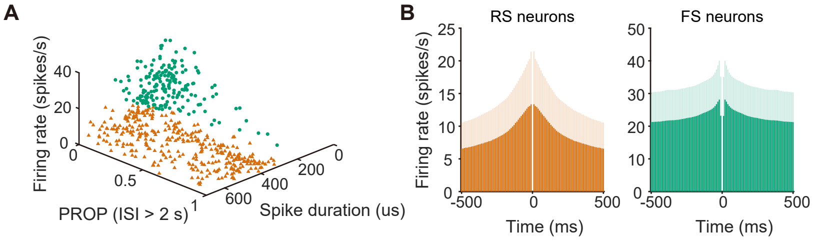


**Figure S4. Properties of RS and FS neurons in the pStr. Related to Figure 2.**
(A) 3D scatter plot of waveform parameters and firing characteristics of each unit, following prior studies[S2,S3]. PROP (ISI > 2 s) was defined as the proportion of total recording time occupied by inter-spike-intervals (ISIs) longer than 2 s (sum of ISIs > 2 s divided by total recording time). Orange triangles and green circles denote RS and FS neurons, respectively.
(B) Autocorrelograms for RS and FS neurons (mean ± s.d. across units).

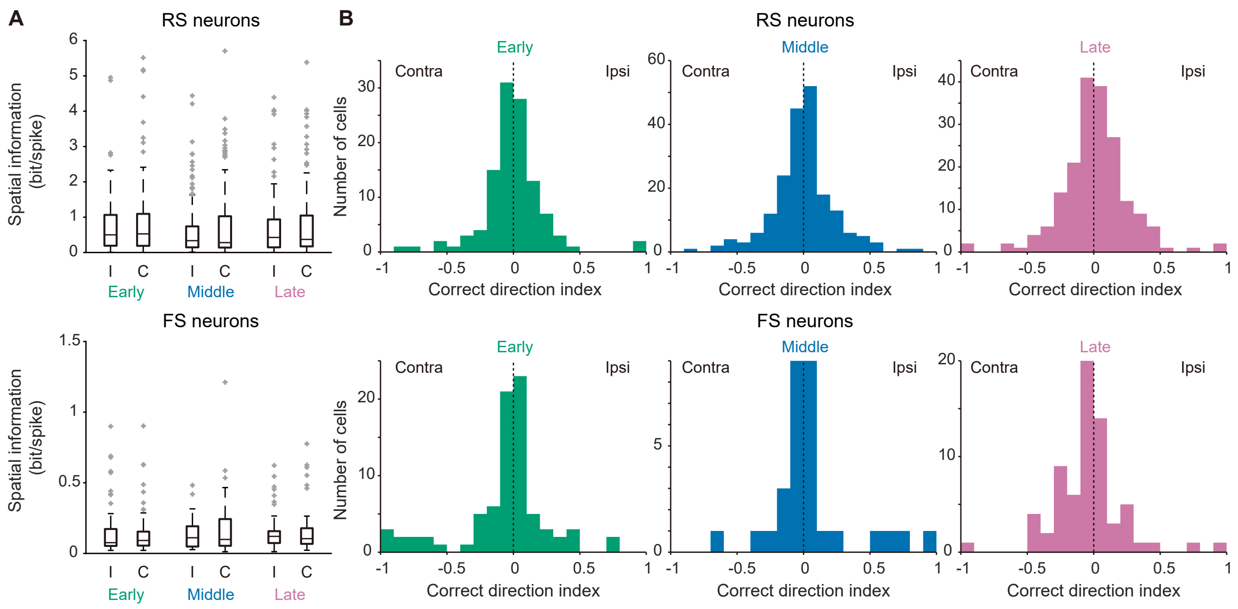


**Figure S5. pStr neuronal activities relative to ipsilateral versus contralateral correct movement directions. Related to Figure 2.**

(A) Spatial information for pStr populations (top, RS neurons; bottom, FS neurons) at each learning stage. I and C denote trials in which the correct movement direction was ipsi- or contralateral to the recording site, resepectively.

(B) Histogram of the correct direction index, calculated as (I - C)/(I + C), where I and C are the average firing rates during traversal of the judgment zone in trials with correct ipsi- and contraleteral movement directions, respectively.

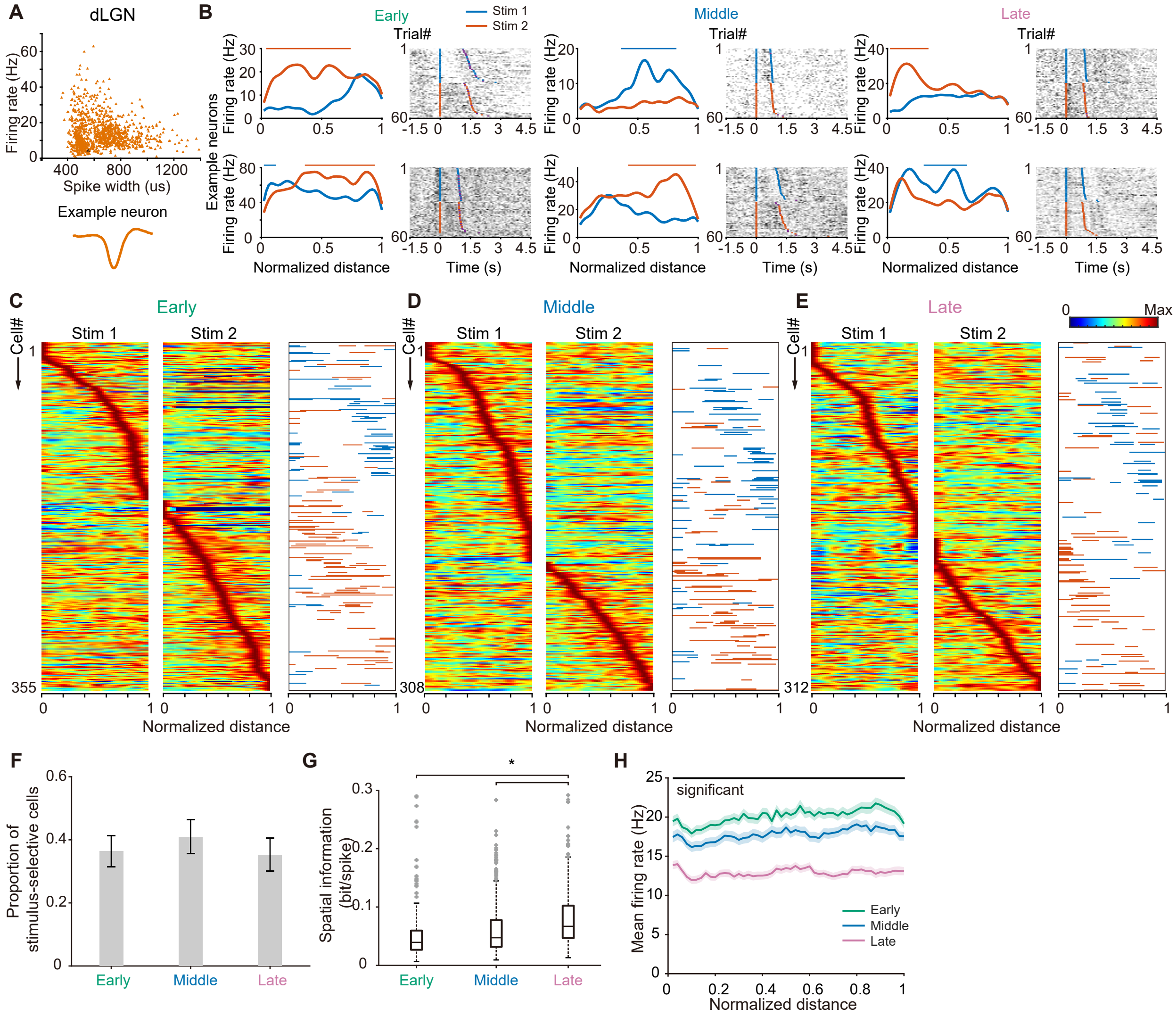

**Figure S6. dLGN activity in the visual discrimination task and its evolution with learning. Related to Figure 2.**
(A) Unit properties in the dLGN. An example waveform is shown below (the corresponding unit is highlighted in black in the top panel).
(B) Firing patterns of representative neurons in the early, middle, and late learning stages. Left, average firing rate as a function of position in the judgment zone for Stim 1 (blue) and 2 (red). Bars above the plots mark positions with significant differences between Stim 1 and 2 ($p < 0.05$, permutation test). Right, spike raster plots as a function of time for each trial sorted by stimulus type. The first blue or red lines represent the time the rats entered the judgment zone and the second lines represent the time the rats exited the judgment zone, followed by the monitors turning gray.
(C-E) Firing patterns of dLGN neurons in Stim 1 (first column) and 2 (second column) trials in the early (C), middle (D), and late (E) learning stages. The third column shows positions with significantly higher discharge rates in Stim 1 (blue) or 2 (red) trials ($p < 0.05$, permutation test).
(F) Proportion of stimulus-selective cells in the dLGN populations at each learning stage. Vertical bars represent the 95% confidence interval (Clopper-Pearson method).
(G) Spatial information in dLGN populations at each learning stage (*: $p < 0.05$, one-way ANOVA).
(H) Average firing rate of dLGN neurons in the early (green), middle (blue), and late (purple) learning stages. Shaded areas represent ± sem. Horizontal bars above the plots represent the positions with significantly higher firing rate between early and late sessions ($p < 0.05$, permutation test).

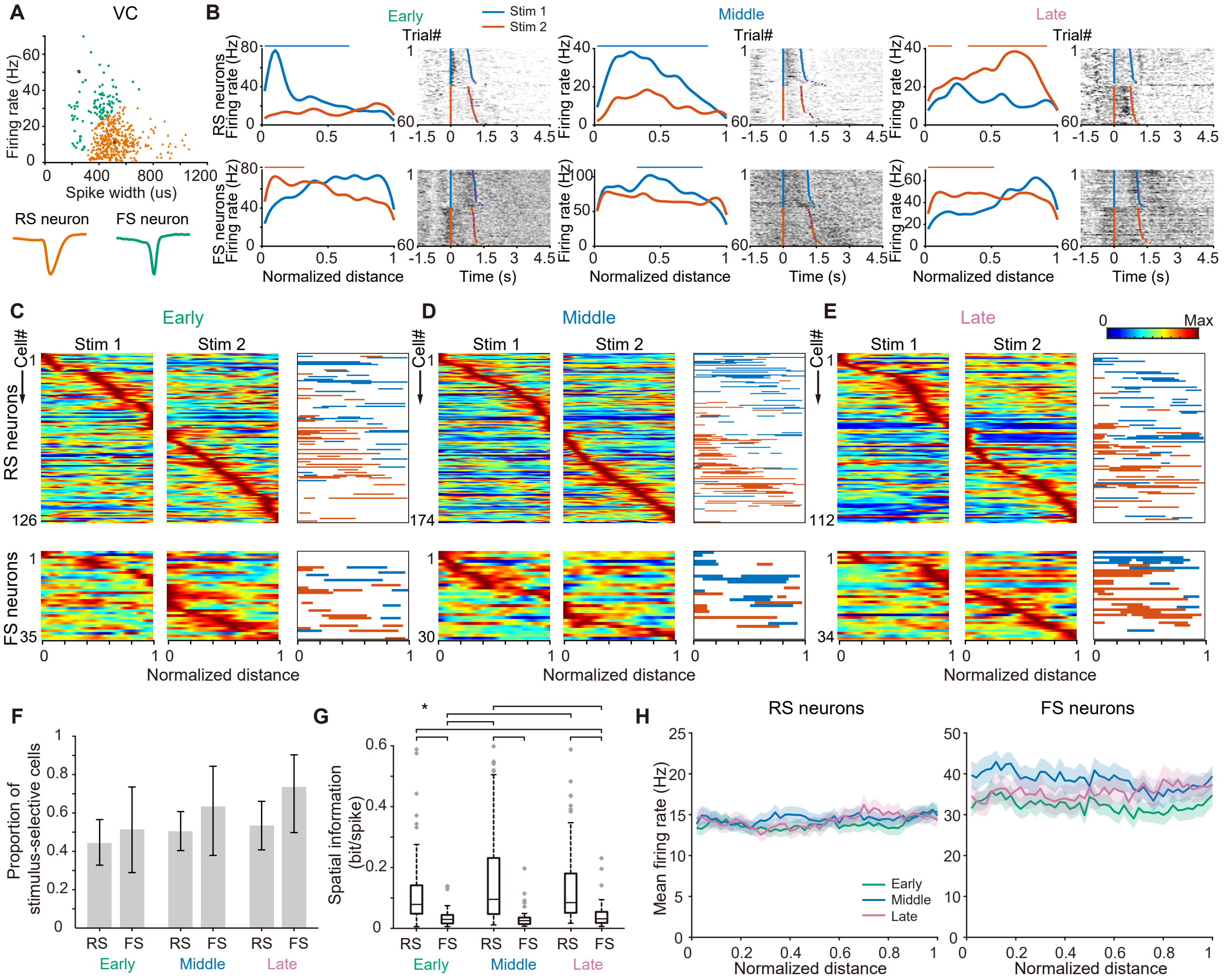

**Figure S7. VC activity in the visual discrimination task and its evolution with learning. Related to Figure 2.**
(A) VC units were classified by spike width and firing rate into regular-spiking (RS; orange triangles) and fast-spiking (FS; green circles) neurons. Example waveforms for each cluster are shown below (the corresponding units are highlighted in black in the top panel).
(B) Firing patterns of representative RS (top) and FS (bottom) neurons in the early, middle, and late learning stages. Left, average firing rate as a function of position in the judgment zone for Stim 1 (blue) and 2 (red). Bars above the plots mark positions with significant differences between Stim 1 and 2 ($p < 0.05$, permutation test). Right, spike raster plots as a function of time for each trial sorted by stimulus type. The first blue or red lines represent the time the rats entered the judgment zone and the second lines represent the time the rats exited the judgment zone, followed by the monitors turning gray.
(C-E) Firing patterns of VC RS (top) and FS (bottom) neurons in Stim 1 (first column) and 2 (second column) trials during the early (C), middle (D), and late (E) learning stages. The third column shows positions with significantly higher discharge rates in Stim 1 (blue) or 2 (red) trials ($p < 0.05$, permutation test).
(F) Proportion of stimulus-selective cells in the VC RS and FS populations at each learning stage. Vertical bars represent the 95% confidence interval (Clopper-Pearson method).
(G) Spatial information in VC RS and FS populations at each learning stage (*: $p < 0.05$, two-way ANOVA).
(H) Average firing rate of VC RS (left) and FS (right) neurons in the early (green), middle (blue), and late (purple) learning stages. Shaded areas represent ± sem.

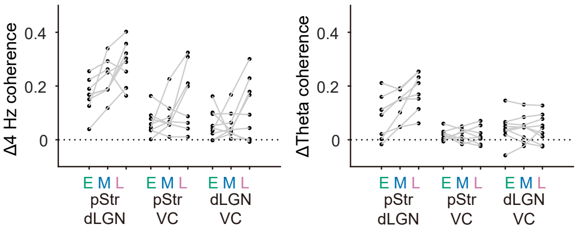


**Figure S8. Wavelet coherence relative to a time-shifted surrogate. Related to Figure 3.**
Δ coherence values (real - surrogate 95th percentile) are shown for 4 Hz (left) and theta (right) oscillations. Black dots represent individual animals at each learning stage, and values above zero indicate that the observed coherence exceeded the level expected by chance. Surrogate data were computed from 100 random LFP time shifts of 0.5 – 2 s.

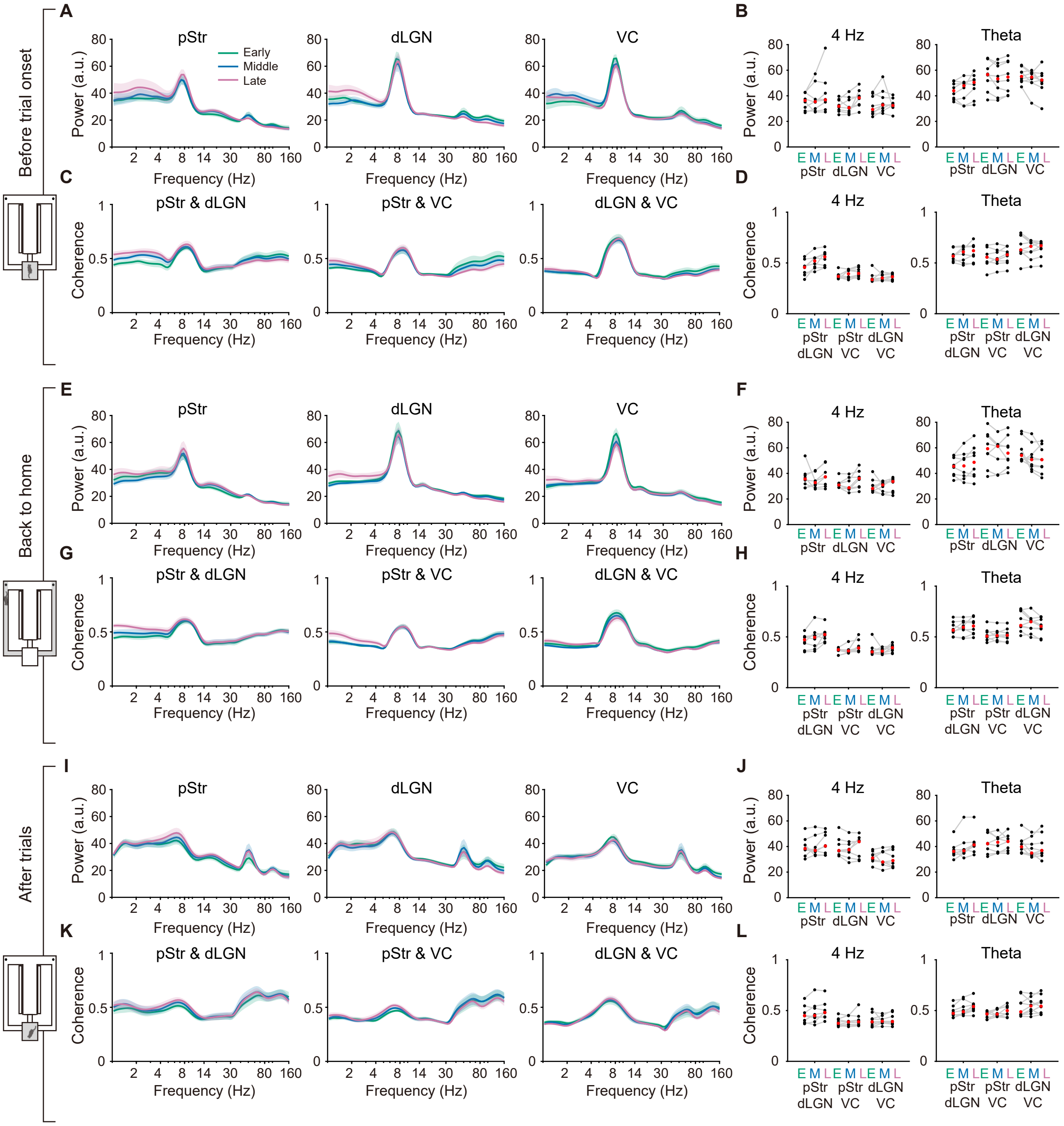


**Figure S9. Wavelet power and coherence during task phases outside the judgment zone. Related to Figure 3.**

(A) Average wavelet power before trial onset (N = 9 rats for pStr and N = 10 rats for dLGN and VC). Green, blue, and purple lines represent early, middle, and late stages, respectively. Shaded areas represent ± sem.

(B) Mean 4 Hz (left, 2-5 Hz) and theta (right, 6-10 Hz) wavelet power before trial onset for each region and learning stage. Black dots represent individual rats and red dots represent group means.

(C) Same as (A), but wavelet coherence.

(D) Same as (B), but 4 Hz and theta wavelet coherence.

(E-H) Same analyses as (A-D) while the rats traversed the side arms back to the home zone.

(I-L) Same analyses as (A-D) after the entry into the home zone.

Neither 4 Hz nor theta power or coherence changed significantly during these phases (one-way ANOVA).

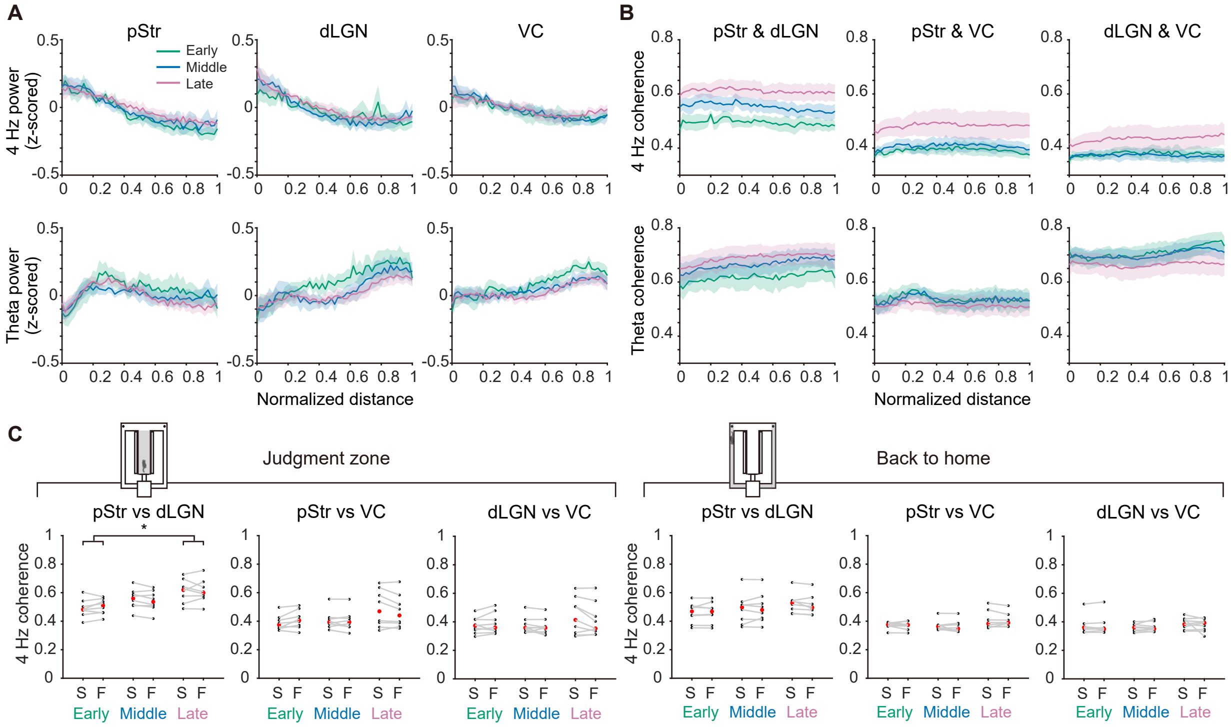


**Figure S10. Spatial and outcome-related dynamics of 4 Hz and theta oscillations during learning. Related to Figure 3.**

(A) Averaged z-scored wavelet power in the 4 Hz (2-5 Hz) and theta (6-10 Hz) bands aligned to normalized distance during the early (green), middle (blue), and late (purple) learning stages. Shaded areas indicate ± sem.

(B) Same as (A) but wavelet coherence.

(C) Averaged 4 Hz and theta wavelet coherence when the rats ran the judgment zone (left) and the side arms back to the home zone (right) in success ('S') and failure ('F') trials in each region pair and learning stage. Black dots represent each rat and red dots represent the mean of rats. *: $p < 0.05$, two-way ANOVA.

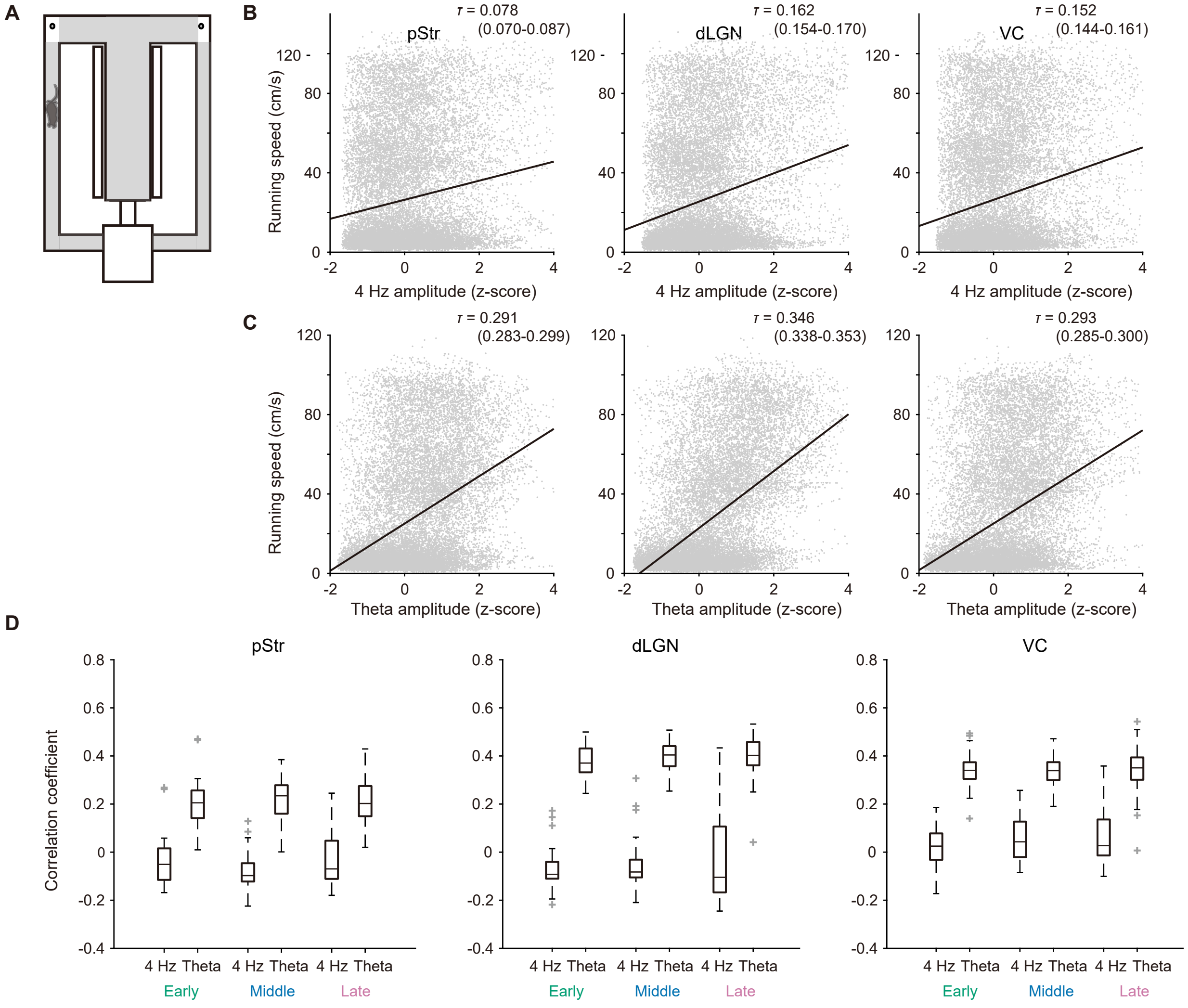


**Figure S11. Theta power correlated with the running speed of rats. Related to Figure 3.**
(A) Kendall's tau coefficients between 4 Hz or theta oscillation amplitude and running speed were calculated for each session and animal. To eliminate the effects of reward consumption, analyses were restricted to periods when the rats were in the passage arms (shaded areas).
(B) Scatter plots of normalized 4 Hz Hilbert amplitude in the pStr (left), dLGN (middle), and VC (right) versus instantaneous running speed in an example session. Each dot represents an instantaneous 4 Hz amplitude and running speed pair. Black lines represent least-squares fits. Tau coefficients and the 95% confidence intervals in the parentheses are shown in the upper-right corners.
(C) Same as (B) but theta Hilbert amplitude.
(D) Distribution of Kendall's tau coefficients between the Hilbert amplitude and running speed at each learning stage. Theta amplitude in all regions showed significant positive correlations with running speed throughout the learning stages, whereas 4 Hz amplitude showed only weak correlations. No significant changes in these correlations across stages were detected (one-way ANOVA).

## Table S1. Recorded units. Related to Figure 1.

| | pStr | dLGN | VC |
|---|---|---|---|
| Early (72 sessions) | 198 | 362 | 170 |
| Middle (108 sessions) | 230 | 318 | 208 |
| Late (195 sessions) | 261 | 315 | 148 |

**Supplemental references**

[S1] Paxinos, G., and Watson, C. (2008). The rat brain in stereotaxic coordinates: Compact 6th edition 6th ed. (Academic Press).
[S2] Yamin, H.G., Stern, E.A., and Cohen, D. (2013). Parallel processing of environmental recognition and locomotion in the mouse striatum. J. Neurosci. 33, 473–484. https://doi.org/10.1523/JNEUROSCI.4474-12.2013.
[S3] Peters, A.J., Fabre, J.M.J., Steinmetz, N.A., Harris, K.D., and Carandini, M. (2021). Striatal activity topographically reflects cortical activity. Nature 591, 420–425. https://doi.org/10.1038/s41586-020-03166-8.
